# Targeted curation of the gut microbial gene content modulating human cardiovascular disease

**DOI:** 10.1128/mbio.01511-23

**Published:** 2023-09-11

**Authors:** Mikayla A. Borton, Michael Shaffer, David W. Hoyt, Ruisheng Jiang, Jared B. Ellenbogen, Samuel Purvine, Carrie D. Nicora, Elizabeth K. Eder, Allison R. Wong, A. George Smulian, Mary S. Lipton, Joseph A. Krzycki, Kelly C. Wrighton

**Affiliations:** 1 Department of Soil and Crop Sciences, Colorado State University, Fort Collins, Colorado, USA; 2 Environmental and Biological Sciences Directorate, Pacific Northwest National Laboratory, Richland, Washington, USA; 3 Department of Microbiology, The Ohio State University, Columbus, Ohio, USA; 4 Department of Internal Medicine, University of Cincinnati, Cincinnati, Ohio, USA; University of Kentucky, Lexington, Kentucky, USA

**Keywords:** methylated amine, MAGICdb, microbiome, atherosclerosis, metagenomics, metatranscriptomics, linear regression, choline, carnitine, trimethylamine

## Abstract

Despite the promise of the gut microbiome to predict human health, few studies expose the molecular-scale processes underpinning such forecasts. We mined over 200,000 gut-derived genomes from cultivated and uncultivated microbial lineages to inventory the gut microorganisms and their gene content that control trimethylamine-induced cardiovascular disease. We assigned an atherosclerotic profile to the 6,341 microbial genomes that encoded metabolisms associated with heart disease, creating the Methylated Amine Gene Inventory of Catabolism database (MAGICdb). From microbiome gene expression data sets, we demonstrate that MAGICdb enhanced the recovery of disease-relevant genes and identified the most active microorganisms, unveiling future therapeutic targets. From the feces of healthy and diseased subjects, we show that MAGICdb predicted cardiovascular disease status as effectively as traditional lipid blood tests. This functional microbiome catalog is a public, exploitable resource, designed to enable a new era of microbiota-based therapeutics and diagnostics.

**IMPORTANCE:**

One of the most-cited examples of the gut microbiome modulating human disease is the microbial metabolism of quaternary amines from protein-rich foods. By-products of this microbial processing promote atherosclerotic heart disease, a leading cause of human mortality globally. Our research addresses current knowledge gaps in our understanding of this microbial metabolism by holistically inventorying the microorganisms and expressed genes catalyzing critical atherosclerosis-promoting and -ameliorating reactions in the human gut. This led to the creation of an open-access resource, the Methylated Amine Gene Inventory of Catabolism database, the first systematic inventory of gut methylated amine metabolism. More importantly, using this resource we deliver here, we show for the first time that these gut microbial genes can predict human disease, paving the way for microbiota-inspired diagnostics and interventions.

## INTRODUCTION

**Fig 1:**
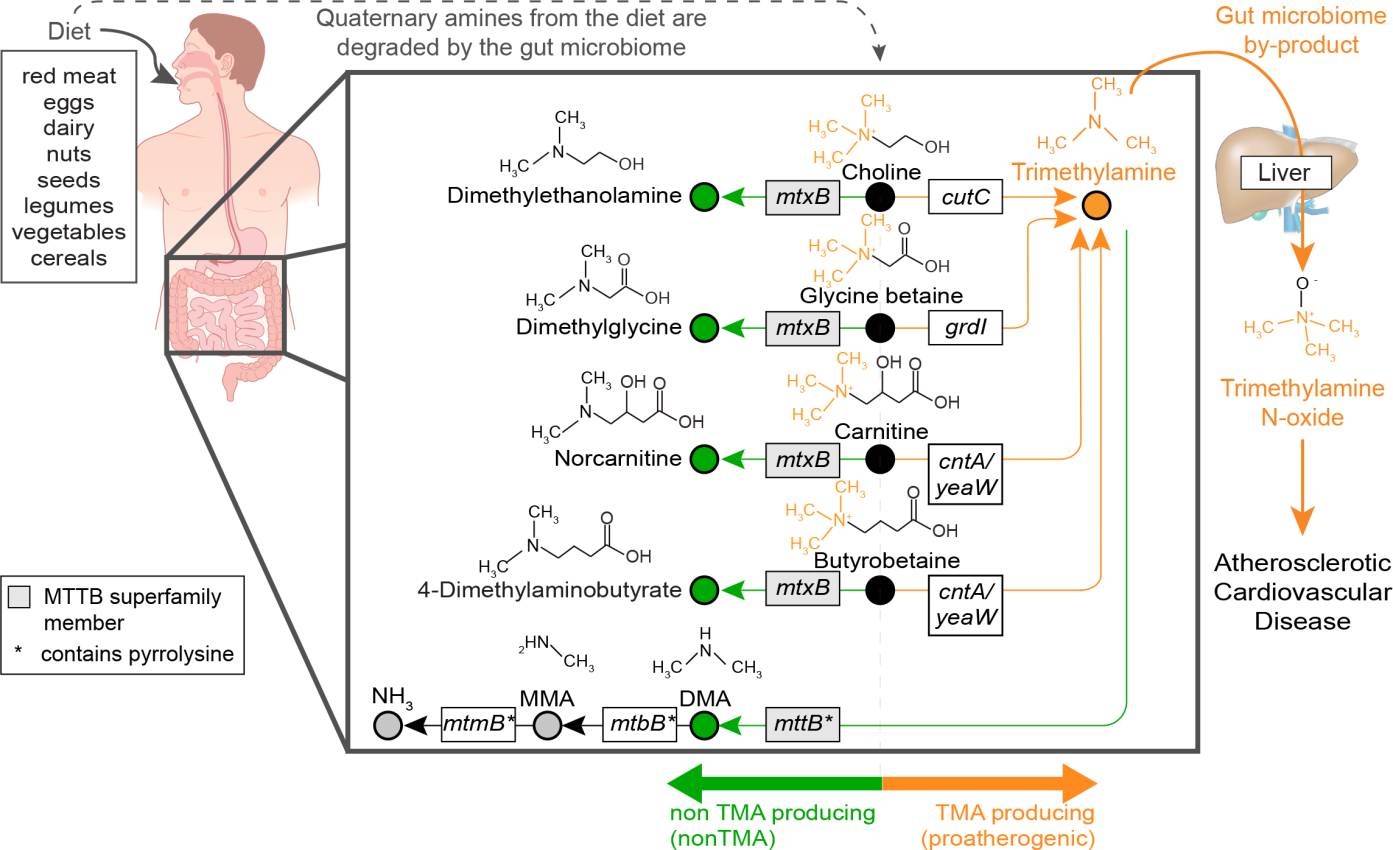
Dietary quaternary amine transformation routes in the gut. Foods in the human diet, including red meat and certain vegetables, have elevated quaternary amines (10). Upon consumption, these compounds travel to the gut where they are degraded by microorganisms. Chemical structures for dietary quaternary amines (black circles) are shown for choline, glycine betaine, carnitine, and butyrobetaine, with the trimethylamine (TMA) moiety of these compounds noted in orange. Microbial proatherogenic conversions (orange arrow) of these compounds yield TMA (orange circle), which is exported to the liver where human enzymes convert TMA to trimethylamine-*N*-oxide, a metabolite that promotes atherosclerosis. Alternatively, microorganisms use two demethylation routes to subvert TMA concentrations (green arrows). In the first, microbial dietary quaternary amine processing does not result in TMA production, but instead yields non-TMA metabolites (green circles) such as dimethylethanolamine, dimethylglycine, norcarnitine, or 4-dimethylaminobutyrate. In the second, TMA is directly demethylated to dimethylamine (DMA, green circle). Sequential demethylations of DMA to MMA (monomethylamine) and MMA to ammonium are also noted by gray circles. For each conversion, the microbial abbreviated gene names are noted in boxes, with the full gene names, reactions, and citations included in Data S1.

Mounting evidence implicates the gut microbiome, the thousands of microorganisms and their gene products residing in the gut, as a critical modulator of human health (1, 2). One of most compelling examples connecting gut microbial metabolism to human disease is atherosclerotic cardiovascular disease (ACVD), which is the leading cause of death globally (3 – 6). In ACVD, gut microorganisms process quaternary amines from protein-rich foods (e.g., eggs, beans, meat) to generate the metabolite trimethylamine (TMA, Fig. 1). TMA, an obligate microbiota-derived metabolite, is absorbed into the blood stream and subsequently transformed by the liver to TMA-*N*-oxide, which promotes ACVD in humans (3, 5). Gut microorganisms also catalyze reactions that reduce gut TMA concentrations, many of these were only recently discovered in the past five years (7, 8). While linkages between the gut microbiota and atherosclerosis are accepted (3 – 5, 9), these new discoveries warrant a holistic inventory of the TMA-producing and -reducing microbial reactions in the human gut.

The microbial biochemistry catalyzing TMA production from quaternary amines is commonly attributed to four routes from dietary choline (11), glycine betaine (12), carnitine (13), and butyrobetaine (14) (Fig. 1, orange). Alternatively, through demethylation reactions, microorganisms can either directly reduce TMA concentrations (15), or act on dietary quaternary amines (7, 8, 16 – 19), or indirectly subvert TMA production (Fig. 1, green). These non-TMA-producing demethylation reactions are catalyzed by enzymes belonging to the same superfamily (MTTB), with the TMA-specific enzymes distinguished by the presence of a pyrrolysine amino acid, which encodes an amber stop codon (16, 20, 21). Despite their capacity to reduce concentrations of disease-causing TMA, these non-TMA routes remain enigmatic due to a lack of sampling these genes from mammalian gut microbiomes.

Collectively the microbial conversions of quaternary amines and their derivatives are here referred to as methylated amine (MA) metabolism. In addition to recent discoveries, more broadly MA metabolism remains poorly characterized from microbiome data sets. Complications include erroneous functional assignment due to homology-based searches (22, 23) or missed annotation due to pyrrolysine gene truncation (15, 20, 21). Beyond annotation, many of the known TMA-utilizing microorganisms, such as methanogens, are rare members in the gut and are often missed because of sampling considerations (24, 25). As a result, MA metabolism in the gut has not been systematically inventoried, hindering our ability to reliably predict and eventually manage TMA-induced atherosclerotic disease.

To address this knowledge gap, we cataloged the proatherogenic and non-TMA-producing gene content from more than 200,000 microbial genomes derived from the human gut (26 – 28), constructing the Methylated Amine Gene Inventory of Catabolism database (MAGICdb). We then provide proof-of-principle demonstrations for this resource, including (i) evidence that quaternary amines can be demethylated to non-TMA products in fecal reactors, (ii) an inventory of the most active TMA-producing and -reducing microorganisms from human cohorts, and (iii) predicting human cardiovascular disease from microbial gene content in feces. This open genomic resource paves the way for disease diagnoses and management from microbiome content, representing a new avenue for the development of therapeutic interventions in precision medicine.

## RESULTS AND DISCUSSION

### MA transformations are a keystone metabolism in the human gut

To inventory the MA content in the gut microbiome, we developed a computational workflow that overcame prior annotation challenges by employing homology and non-homology approaches (Fig. S1). We first identified homologs within each of the seven gene types and then followed two distinct curation paths (i) for the nonatherogenic superfamily members (*mtxB*, *mttB*) and other demethylating genes (*mtmB*, *mtbB*) and (ii) for proatherogenic members (*cutC*, *cntA*/*yeaW*, *grdI*). Following manual curation of these genes, the microbial genomes were defined as proatherogenic (TMA-producing), non-TMA producing, or both based on their collective gene content.

**Fig 2:**
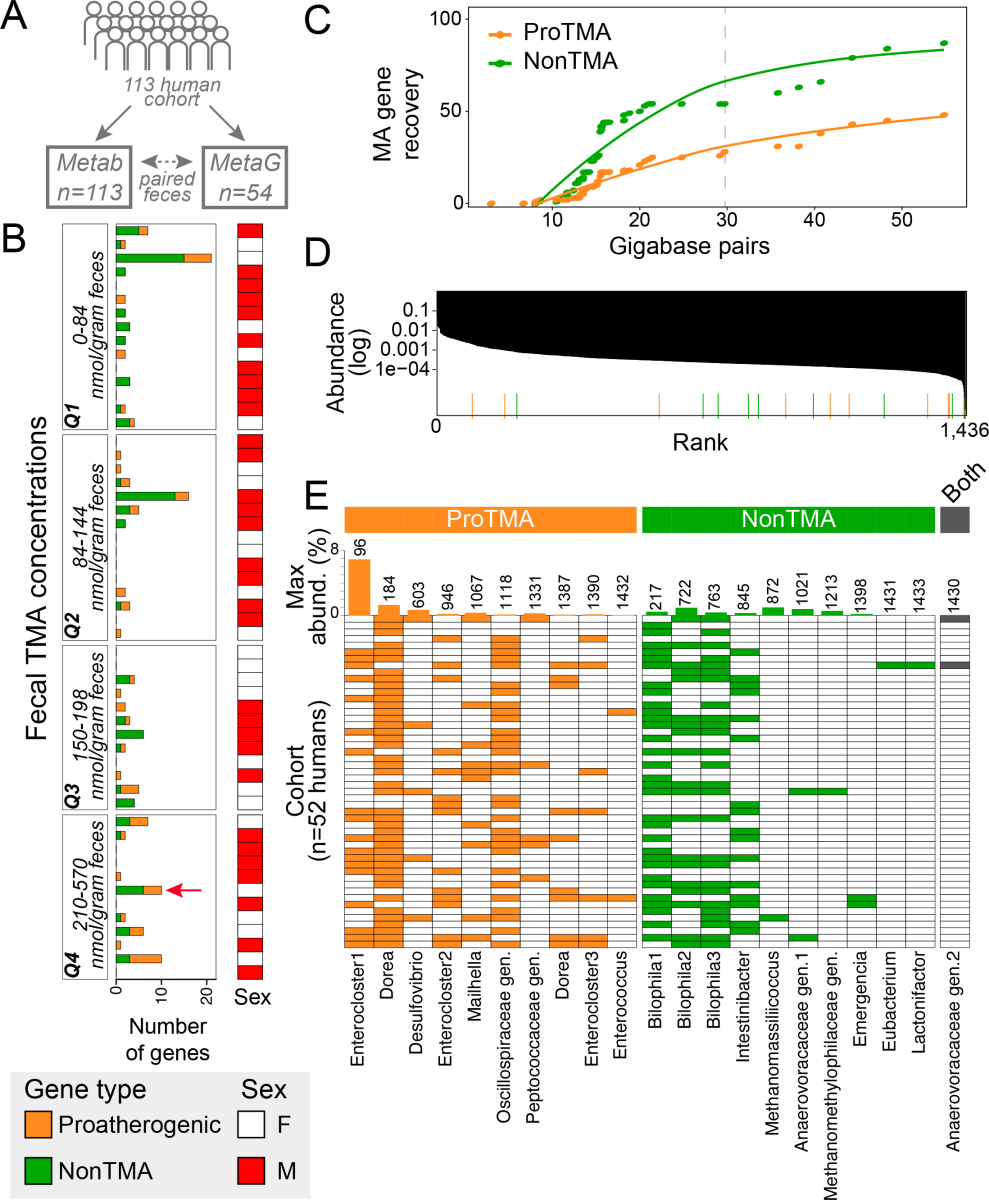
Microbial methylated amine utilization is encoded by rare members with differential occupancy sampled across a human cohort. (**A**) A 113 human cohort study resulted in metabolite analysis (Metab) of fecal TMA concentrations, which were assigned to quartiles (Q1 to Q4) based on concentration. Using these quartiles, 54 samples were selected for metagenomics (MetaG), with at least 12 samples chosen from each quartile. (**B**) Quantification of the proatherogenic (orange) and non-TMA (green) genes inventoried in each fecal metagenome, organized by quartile, with quartile ranges noted in the left panel and sex of the subject noted in the right panel. Red arrow denotes the fecal sample chosen for quaternary amine amendment in Fig. 4. (**C**) MA discovery curve denotes the number of new genes recovered with increased sequencing depth. The dashed line indicates the plateau of new MA gene recovery. (**D**) A rank abundance curve of the average relative abundance of 1,436 MAGs (*y*-axis) and their average rank (*x*-axis) in each sample sequenced as part of this cohort. The average ranked relative abundance of MA-containing genomes is highlighted by colored bars along the *x*-axis. (**E**) The presence (filled) and absence (white) of MA-containing MAGs, with the bar graphs at the top reporting the maximum relative abundance and average rank (numbers out of 1,436) of each genome. In panels B–E, colors correspond to proatherogenic (orange), non-TMA producing (green), or both (black), based on MA content as defined in Fig. 1.

We first performed a human cohort study of 113 individuals (Fig. 2A; Fig. S2), to enable us to link fecal TMA concentrations to microbial gene content. We applied our computational workflow to 54 fecal metagenomes that spanned quartiles of fecal TMA concentrations derived from our cohort (Fig. 2B). To sample rare microbial members, these fecal metagenomes were sequenced up to 55 Gbp/sample (mean of 18 Gbp/sample, Data S1), resulting in deeper sequencing than is commonly used in gut metagenome studies (~4 Gbp/sample) (29 – 31). We show that a metagenome sequence depth of more than 35 Gbp recovered nearly double the amount of MA genes than the traditional 4 Gbp (Fig. S3). At a cumulative sequencing depth of 775 Gbp (i.e., 75% of our total sequencing) the MA gene discovery rate plateaued (Fig. 2C). Beyond sequencing depth, gene recovery was also enhanced by the number of individuals sampled, suggesting this metabolism may be variably dispersed across humans (Fig. 2D and E). These analyses reinforce the importance of considering gene abundance and cohort distribution when designing experiments to target specific metabolisms from a complex microbiome like the gut.

From our cohort we sampled 153 MA genes (135 unique) with 41% and 59% of the genes given proatherogenic or non-TMA assignments, respectively (Data S1). We found no considerable relationship between gene content or TMA concentrations with host sex, body mass index, or lifestyle (Fig. S2). Consistent with their physiological roles (Fig. 1), the relative abundance of the proatherogenic *cntA*/*yeaW* gene was correlated with higher fecal TMA concentrations (Fig. S2C and S3B), while the relative abundance of the non-TMA *mttB* and *mtxB* genes was associated with lower TMA concentrations (Fig. S3). More importantly, while neither the proatherogenic nor the non-TMA-summed relative abundances were on their own able to predict fecal TMA concentrations, together their cumulative profile was able to significantly explain measured TMA concentrations (Fig. S3). While deduced from a small-sized cohort, this early data support the notion that the comprehensive MA functional content may have explanatory relevance for the atherosclerotic status in humans, a relationship we test more comprehensively subsequently.

Across the cohort we reconstructed 2,447 high- and medium-quality microbial metagenome-assembled genomes (MAGs) that were dereplicated into 1,436 genomic representatives (Data S1). These representatives included 21 MA gene-containing genomes, which were nearly equally classified as proatherogenic and non-TMA producing (Fig. 2D). Only a single genome, a member of a novel genus in the Anaerovoracaceae, had both TMA-forming and -depleting genes. Confirming our earlier suspicions, microbial members that encoded MA genes, especially the less-studied non-TMA types, were some of the rarest members in the fecal community and were unevenly distributed across the cohort (Fig. 2D and E).

Highlighting this variable distribution across the cohort, the most-dominant MA-encoding genome was a proatherogenic *Enterocloster,* which was the 96th most-abundant member detected in a third of individuals (Fig. 2E). The second most-abundant member, a proatherogenic *Dorea*, was detected in nearly every human sampled. The three most-dominant non-TMA-producing genomes were all strains of *Bilophila*, which relative to the TMA-producing genomes were far less abundant (Fig. 2E). Taken together our data suggest that despite being encoded by rare and variably distributed members, the gene relative abundance explains host fecal TMA concentrations (22% variability in TMA concentrations explained, Fig. S3D). Here, we propose that MA metabolism is gut keystone metabolism (extending the idea from keystone species [32, 33]), defined as a process with a disproportionate effect on host physiology despite a low relative abundance in the gut microbiome.

### Curation of MA metabolism from over 200,000 microbial genomes

**Fig 3:**
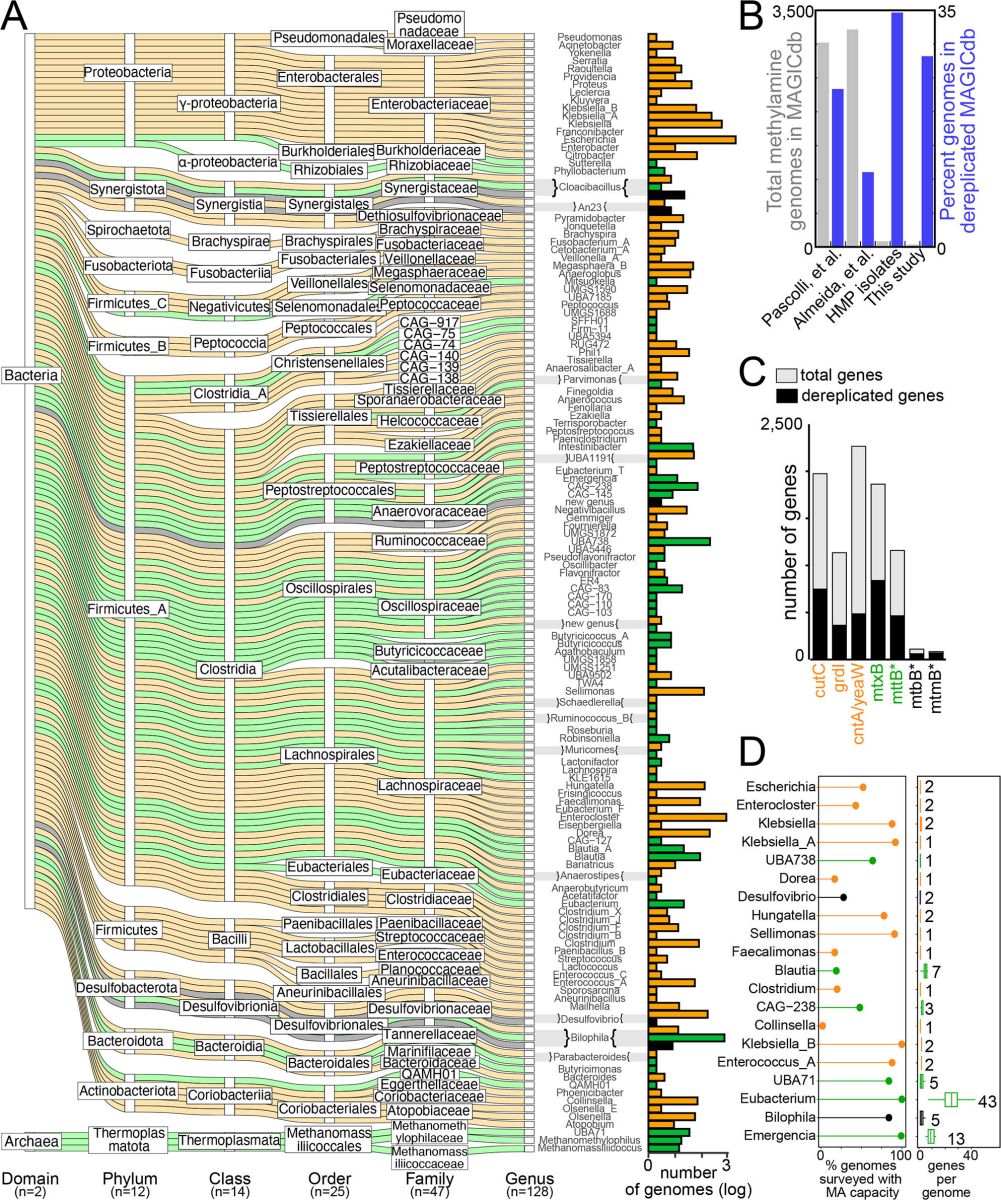
MAGICdb indexes the TMA-relevant gene and genome content in the human gut microbiome. Throughout this figure TMA classifications are based on MA gene or genomic content as outlined in Fig. 1, with colors denoting TMA status as proatherogenic (orange), non-TMA producing (green), or both (black). (**A**) Alluvial plot shows the taxonomic assignment of the 6,341 genomes that encode MA potential in MAGICdb. Alluvia are colored by MA genome content and TMA classification noted by coloring. The total number of genomes and their TMA classification(s) are summarized for each genus as a bar chart. (**B**) The origin of the 6,341 genomes in MAGICdb (gray bars) and the percentage of genomes that remained in MAGICdb after dereplication (blue bars). (**C**) At the gene level, a stacked bar chart reports the total (gray) and dereplicated (black) genes in MAGICdb, with asterisk indicating genes with a pyrrolysine amino acid. (**D**) The top 20 genera represented in MAGICdb and their TMA classification. For the genera with the most genomes sampled in the MAGICdb, the dot plot shows the percentage of genomes surveyed within a genus with the capacity for MA metabolism, while the box plots indicate the mean number and range of MA genes per genome within a genus. For each genus, the maximum number of MA genes in a genome is reported.

To create a robustly sampled MA genome and gene resource, we employed our computational workflow on 237,273 bacterial and archaeal MAGs from publicly available gut collections (26, 27). We also mined 700 bacterial genomes from gut microorganisms cultivated as part of the Human Microbiome Project (28). In total, we analyzed the MA genes from 238,530 microbial genomes acquired from cultivated and uncultivated microorganisms (Fig. 3A and B). Showing the value of each of these data sets, the large-scale MAG compendiums provided the most genomes, while our cohort study-derived MAGs and the microbial genomes from cultivated representatives provided a larger percentage of higher quality genomes that were maintained in the dereplicated database (Fig. 3B).

Mining this genome content, we created MAGICdb, which included (i) a gene data set with unprecedented sampling of these disease-relevant genes and (ii) the corresponding linked genome data set offering organismal context for MA metabolisms. MAGICdb contains 6,341 genomes encoding 8,721 MA genes (Fig. 3A; Data S2). Within the MAGICdb, the proatherogenic and non-TMA gene richness was nearly equivalent (1,597 and 1,434, respectively) with *cutC* (choline TMA lyase) and *mtxB* (non-pyrrolysine methyltransferase) being the most dominant types sampled, respectively (Fig. 3C). Considering the unique genes only, MAGICdb sampled up to 12-fold more genes compared to prior reports (22, 24, 34 – 37). This expansion of MA gene diversity was attributed to the vast number of genomes collected from nonreference-based gut metagenome samples, rather than only relying on genomes from cultivated microorganisms like most prior analyses.

Within the MAGICdb, MA-encoding members belonged to 1 archaeal and 11 bacterial phyla, or half of the phylum-level lineages surveyed (Fig. 3A). This metabolism was found in less than 3% of the microbial gut genomes surveyed, indicating that even when scaled to a larger data set this is a specialized metabolic capacity in the gut microbiome. Here, we discovered the first MA-containing genomes within the phyla *Spirochaetota* (12 MAGs exclusively proatherogenic) and *Synergistota* (59 MAGs with proatherogenic and non-TMA-producing members). We also extended this metabolism to 88 gut genera that, prior to our survey, were not recognized as playing a role in gut MA transformations (Data S2). This novelty sampled in MAGICdb documents the disease-causing or -ameliorating gene reservoir that was previously untapped within the gut microbiome.

Analysis of the TMA classifications across taxonomic levels revealed that all the Archaeal genomes were non-TMA producing. The same cohesive phylogenetic clustering of MA functionality was not observed for the bacteria at higher levels such as class but was observed at finer taxonomic levels such as genera. In fact, 89% of the 125 bacterial genera were exclusively proatherogenic (*Enterocloster*, *Citrobacter, Escherichia*) or non-TMA producing (*Eubacterium, Blautia*) (Fig. 3A). The remaining genera were classified as both, either because a single genome contained both specializations or because a genus contained multiple genomes with contrasting specializations. This heterogeneity is best exemplified in *Bilophila* (in the phylum Desulfobacterota), where a majority of the 711 *Bilophila* genomes were non-TMA producing (97%), 12 MAGs were exclusively proatherogenic, and 7 MAGs encoded both capabilities. It is possible that since these analyses were performed on draft genomes of variable completion some MA content could be missed. However, since 50% of the dereplicated genomes (*n* = 1,092) in MAGICdb were >90% complete, and classifications were validated for taxonomic consistency within a genus, we consider misclassifications due to unsampled genes less likely.

This genome-wide context is a clear strength of our paired gene and genome databases over prior single-gene studies. For example, other genera besides *Bilophila* (e.g., *Desulfovibrio* and a novel genus in Anaerovoracaceae) encoded the ability to produce TMA then subsequently utilize this TMA (Fig. 3D). This concept of a zero-summed game would have been missed if each gene was sampled independently. This finding underscores the value of sampling the entire gene repertoire within a genomic context when identifying microorganisms for possible therapeutic strategies such as probiotics.

Since TMA classification largely followed taxonomic lines, it is tempting to want to assign these metabolic roles from taxonomic data alone, as is often done in 16S rRNA amplicon studies in the gut. However, our analyses underlie the danger in doing this, as these metabolisms are not universally encoded by all genome representatives within a genus. For example, of the genomes surveyed, only 51% of the exclusively proatherogenic *Escherichia* and 19% of the non-TMA-producing *Blautia* genomes sampled encoded MA metabolism (Fig. 3D). These analyses reveal the likelihood for falsely reporting an association or metabolic capacity from taxonomic content alone.

In summary, MAGICdb is a high-quality catalog of the TMA-modulating genes and genomes that are harbored in the human gut. This curated resource will substantially enhance the sampling precision and efficiency of future microbiome studies. For instance, we show that many of these genomes are rare and not evenly dispersed across humans, thus having a higher likelihood of being missed without cultivation or deep metagenomic sequencing. This extensively sampled reference database can now be used as “bait” to capture this metabolism from less deeply sequenced samples, increasing the “mappability” or recovery of reads for this functional gene content from samples where they would not have assembled. In addition, mapping is a far less computationally intensive process, where users can take advantage of our expertly curated indexing to rapidly annotate this gene content in their data sets.

To demonstrate the useability of MAGICdb in this format, this resource was used in three case studies. We use MAGICdb to map gene expression data from our test fecal reactors and two previously published human cohort studies, illuminating the microorganisms actively shaping TMA concentrations in the gut. We also use this resource to recruit gene content in previously published ACVD cohort, demonstrating the efficacy of MAGICdb for disease diagnosing relevance in humans.

### Quaternary amine conversions are an emergent property of the gut microbiome

**Fig 4:**
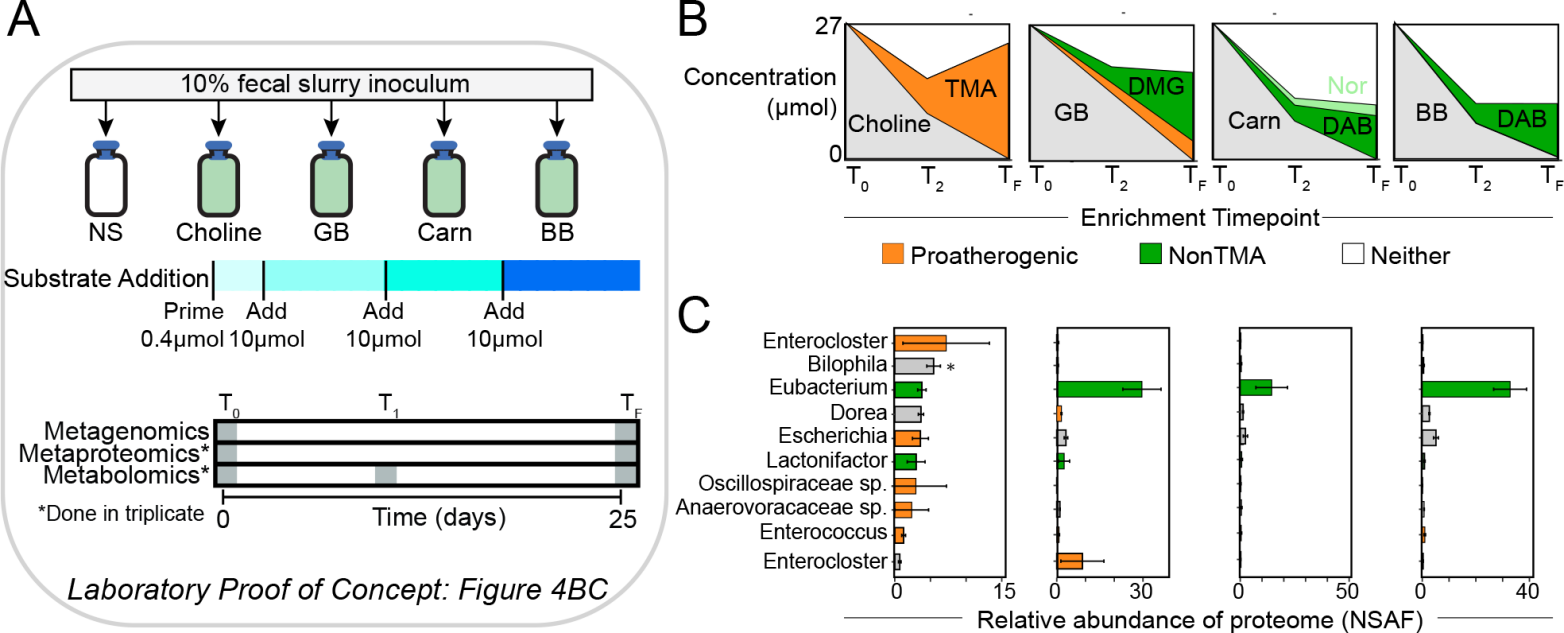
Fecal reactors stimulated with quaternary amines demonstrate MAGICdb contains microorganisms capable of MA transformations. (**A**) Schematic of fecal reactor study design. Fecal inoculum was provided by an individual in our cohort (see Fig. 2B, red arrow) and stimulated separately with each of the four quaternary amines at the dosing shown. Paired multi-omics collected at the beginning and end of the experiment indicated putatively active MA metabolizing microorganisms. (**B**) Area plots show MA metabolite concentrations in the reactors over time with the curve colored by quaternary amine substrate added (gray) and the microbially produced proatherogenic metabolite TMA (orange) or nonatherogenic metabolite(s) (green) noted. TMA, trimethylamine; DMG, dimethylglycine; Carn, carnitine; Nor, norcarnitine; DAB, dimethylaminobutyrate; BB, butyrobetaine. (**C**) The bar chart shows the relative proportion of the metaproteome uniquely assigned to a MAGICdb genome. Microbial bars are colored based on MA metaproteome detection with those potentially contributing to a proatherogenic (orange) or non-TMA producing (green) response denoted. Bars colored in pale gray are genomes that encode MA potential and recruit peptides, but the MA gene content was not expressed under the specific laboratory condition(s). The entire fecal microbial community metaproteome data set is included (Data S3).

Previously active MA transformations by gut microorganisms were only demonstrated using pure cultures, thus the cooperative and competitive processing of quaternary amines and their collective contributions to TMA output remain poorly resolved. To address this knowledge gap, we used reactors to individually dose the same fecal microbial community with each quaternary amine (Fig. 4A). We used MAGICdb to profile the microbial community metaproteome and paired this to quantification of MA metabolites over time.

For both the community-wide metaproteome and metabolite profiles, replicates within each treatment were congruent; however, each quaternary amine resulted in statistically different active microbial communities (Fig. S4). Metabolite quantification revealed that only choline and glycine betaine resulted in a proatherogenic response, with choline exclusively converted to TMA and glycine betaine resulting in both demethylated and TMA metabolites (Fig. 4B). Alternatively, carnitine and butyrobetaine stimulation exclusively produced a nonatherogenic response, a finding reflecting the anoxic reactor conditions that restricted the oxygen-requiring proatherogenic monooxygenases (CntA, YeaW) (13, 14). In summary, we provide the first metabolite and proteomic evidence that these non-TMA-producing reactions can be competitive with TMA-producing reactions using quaternary amines as a substrate.

The proatherogenic response was mediated by a diverse set of microorganisms compared to the nonatherogenic response. Proteomic evidence for TMA production included members of *Enterocloster*, *Dorea*, and *Enterococcus,* as well as newly discovered MAGICdb lineages in the Anaerovoraceae and Oscillospiraceae. In contrast to the proatherogenic response, the demethylation of quaternary amines was mediated by two genomes belonging to the genera *Lactonifactor* and *Eubacterium*, with 3 and 11 *mtxB* genes expressed, respectively. This, to our knowledge, is the first implication for members of the genus *Lactonifactor* in thwarting TMA concentrations in the gut. *Eubacterium*, on the other hand, is the model microorganism for quaternary amine demethylation (Data S1). Interestingly, we observed that a single *Eubacterium* MtxB was expressed in all quaternary amine treatments, suggesting this single enzyme demethylated all substrates. Validating this supposition, this laboratory-identified sequence was 99% similar to a recently biochemically characterized enzyme purified from *Eubacterium limosum* that demethylated butyrobetaine, carnitine, and glycine betaine (7). Our metabolite and metaproteome findings support a growing body of literature that *Eubacterium* are highly specialized for using quaternary amines and producing non-TMA metabolites in the gut, representing an ideal target for probiotic-based therapeutics.

These metaproteome and metabolite analyses also allowed us to contextualize the impacts of this metabolism more broadly on the gut ecosystem. For instance, the concentration of short-chain fatty acids (SCFA, e.g., acetate, butyrate, propionate) increased across all quaternary amine-amended reactors (Fig. S5). Our metaproteomics data demonstrated that organisms actively utilizing quaternary amines also expressed genes to produce SCFAs (Data S3). Given that SCFAs regulate colonocyte energy balance, gut hormone homeostasis, and diabetes (38, 39), understanding MA metabolisms can have other important health outcomes beyond cardiovascular disease.

This genome context also demonstrated that quaternary amines were metabolized using a variety of energetic strategies. We show that proatherogenic microorganisms use quaternary amines to support anaerobic respiration with fumarate or sulfite as electron acceptors or obligate fermentation (Data S3). The non-TMA-producing *Lactonifactor* and *Eubacterium* genomes were inferred to be using these substrates to support an obligatory fermentative lifestyle. Of the 10 genomes expressing proatherogenic and non-TMA-forming genes, *Eubacterium* is the only MA specialist, as all others coexpress glycoside hydrolases, such that we cannot rule out concomitant carbohydrate use. Differences in ATP gained from various MA metabolisms will likely impact microbial biomass production and TMA conversion rates, and thus may warrant further investigation through metabolic modeling of key taxa for translation to microbiota-based therapies.

Together these community-focused analyses reveal that quaternary amine conversions are an emergent property of the microbiome and cannot be fully evaluated by single-gene surveys or outcomes from pure culture experiments. For example, the net result of several members of the microbiome acting on the same substrate with differential metabolite outcomes can only be identified in mixed consortia and not in single isolate experiments. Thus, future engineering of the gut microbiome to control TMA concentrations will need to account for differences in energetics, the metabolic plasticity within an organism, and exchanges between microorganisms.

### MAGICdb illuminates TMA-modulating enzymes previously obscured in human cohorts

**Fig 5:**
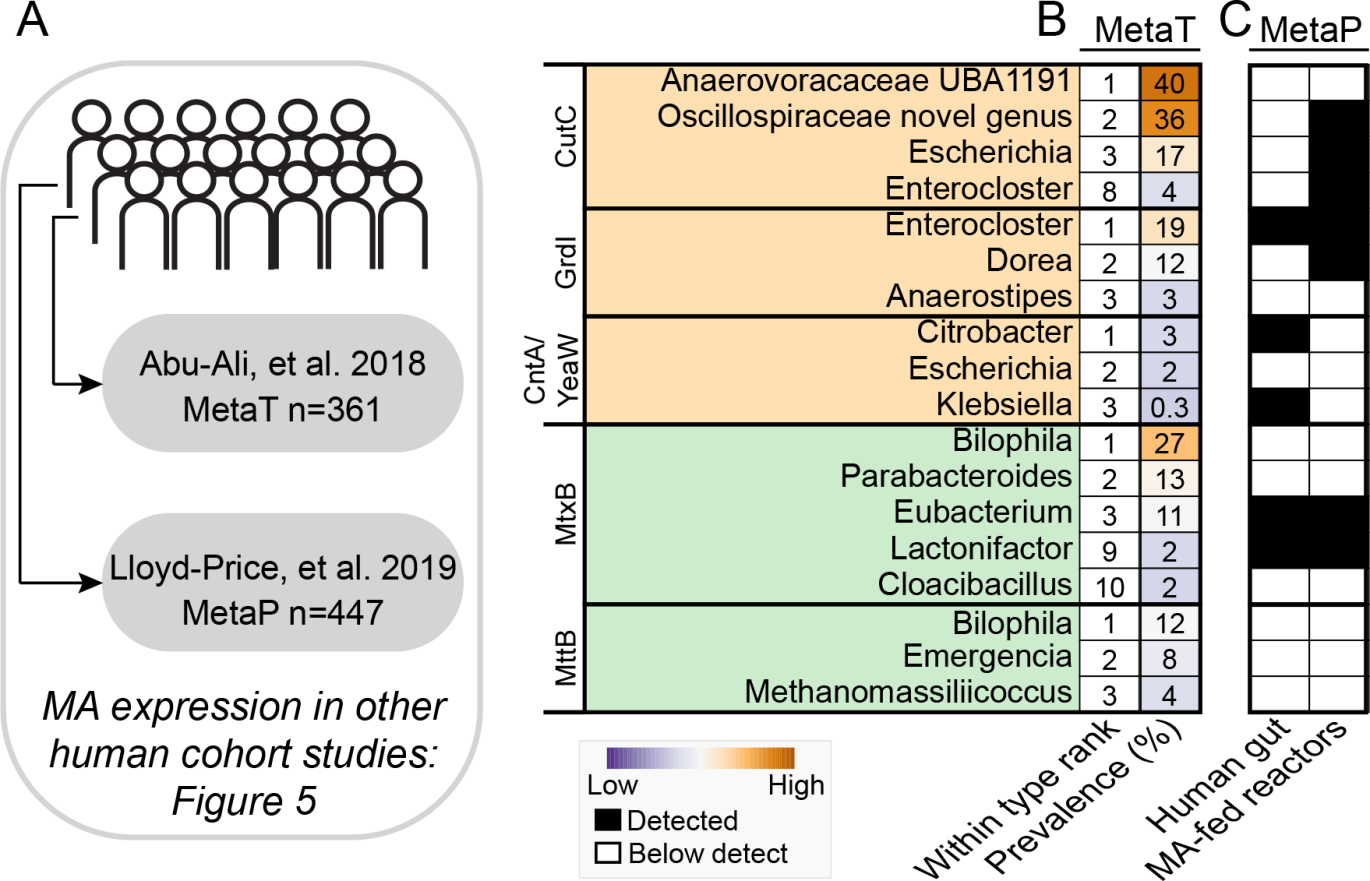
MAGICdb uncovered active microbial members and assigned their metabolic MA roles from *in vivo* human fecal analyses. (**A**) Schematic showing the use of MAGICdb to recruit expression data from 361 fecal metatranscriptomes (MetaT) collected from a human cohort of 96 individuals and from 447 fecal metaproteomes (MetaP) collected from a human cohort of 75 individuals. (**B**) For each MA gene type, the top three genera with the highest summed gene expression are reported, with some selected lower ranking but genera active in metaproteomes also reported. For each genus within gene ranking (one being most expressed) and the cohort prevalence (percentage of metagenomes where gene expression was detected) is quantified. (**C**) We next compared if these genes were also expressed in metaproteomic data sets derived from our reactors (Fig. 4) and *in vivo* from a previously published study (41). Shared gene expression data across studies is reported as presence (black) and absence (white).

To further extend the relevance of this MAGICdb resource, we mapped human fecal metatranscriptomic and metaproteomic data from two published cohorts (40, 41) and found MAGICdb genes were expressed in 82% of 361 metatranscriptomes and in 58% of 447 metaproteomes (Fig. S6). Using the metatranscriptome data, we identified the most highly expressed genes for each of the MA gene types and recorded their prevalence (% of samples detected) and mean relative abundance across the cohort (Fig. 5). We then compared whether these genera that were active in the metatranscriptome analyses had similar gene expression in metaproteomes from a previously published cohort and our fecal reactors. Our goal for analyzing these multitude of data types, fecal sources, and sampled conditions was to constrain the thousands of genomes in MAGICdb to a most wanted list of the most active microorganisms in the human gut, identifying lineages that could represent potential therapeutic or diagnostic targets.

Collectively these proatherogenic findings expanded TMA production beyond the Gammaproteobacteria (e.g. *Escherichia*, *Klebsiella*, *Citrobacter*), which are well documented to produce TMA, to also include members of the class Clostridia (e.g. *Enterocloster*, *Dorea*). Notably, a *Dorea* genome with the same MA functionality was the second most-dominant in our human cohort and detected in 86% of individuals (Fig. 2D). Beyond organisms with cultivated representatives, the cohort gene expression analyses and our fecal reactor data demonstrated that novel members of Anaerovoraceae and Oscillospiraceae (both families in the Clostridia) were responsible for TMA production from choline. While MAGICdb revealed that some members of this Anaerovoraceae genus could encode nonatherogenic genes (Fig. 2D and 3B), our combined *in vivo* and *in vitro* expression data suggest a stronger proatherogenic role might be likely. While today there is limited progress in designing specific microbiota eradication techniques, our coordinated analyses reveal likely targets for precision interventions or diagnostic atherosclerotic biomarkers.

**Fig 6:**
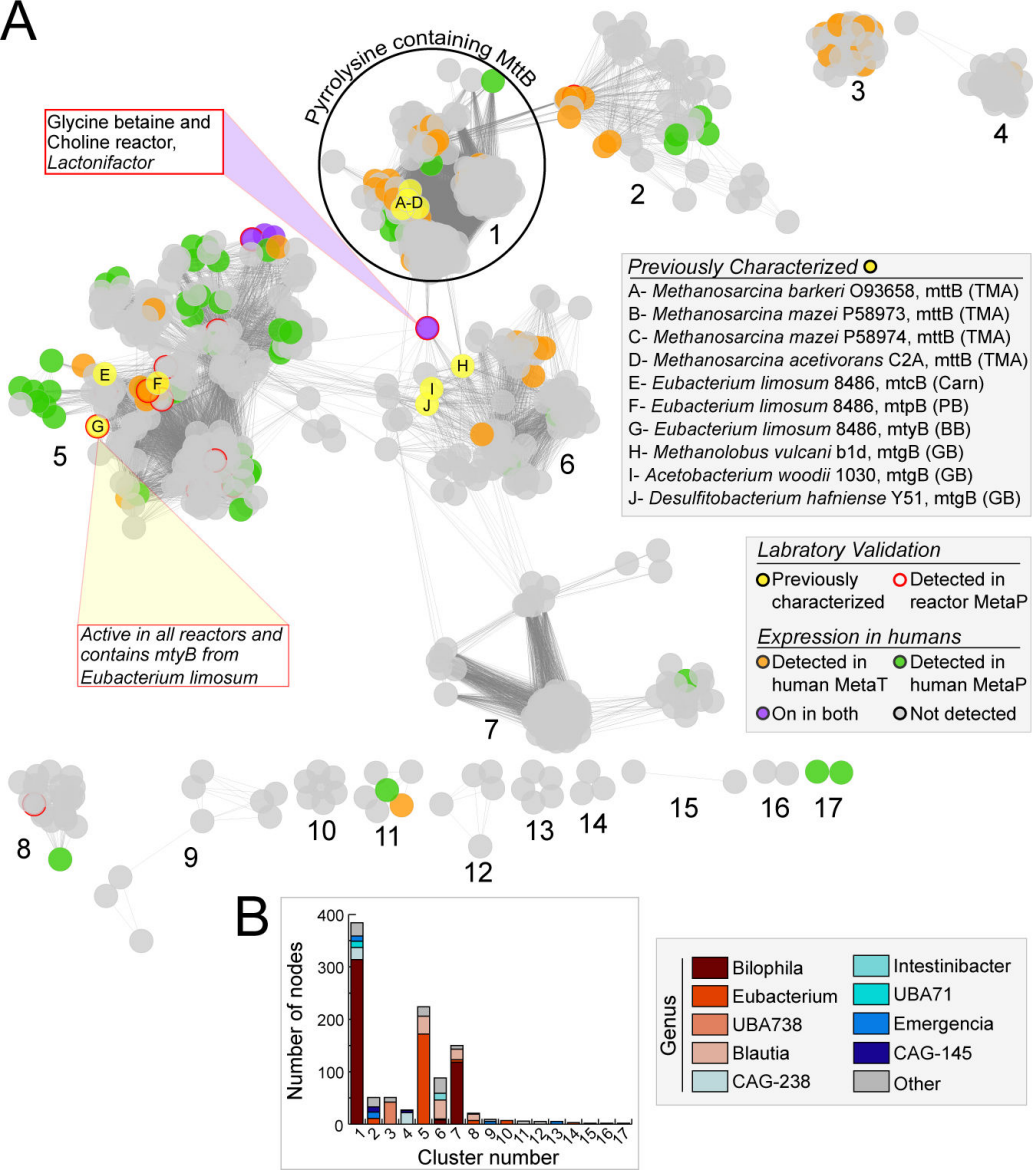
Taxonomy, prevalence, and expression of the MTTB superfamily in MAGICdb. (**A**) Sequence similarity network (SSN) of the MTTB superfamily within MAGICdb with each of the 1,031 nodes (colored dots) representing one or more amino acid sequences (>99% identity) connected by an edge if the pairwise amino acid sequence similarity is >80%. Nodes are colored to represent gene products that were previously biochemically characterized (yellow-filled circles) or recruited to MAGICdb from publicly available microbial gene expression data in feces collected from two large human cohort studies, with metatranscriptome (orange-filled circle), metaproteome (green-filled circle), or both (purple-filled circle) data sets. Nodes with a red outline were expressed in our fecal laboratory metaproteomic data. Previously biochemically characterized MTTB superfamily members are labeled A–J. For these characterized enzymes the microorganisms and preferred substrate are reported in the shaded box with trimethylamine (TMA), carnitine (Carn), proline betaine (PB), butyrobetaine (BB), and glycine betaine (GB) noted. One yellow node, labeled “G,” contained a characterized enzyme from *Eubacterium limosum* ATCC 8486 that was >99% similar to a sequence recovered from a MAG reconstructed here that was expressed in our fecal reactors. (**B**) Stacked bar chart shows the genus-level content of each cluster within the SNN.

One of the most significant findings of MAGICdb was our vast expansion of the non-TMA-producing microbial enzymes and the microorganisms that encode them. A sequence similarity analyses of the 3,022 MTTB superfamily genes in our database resulted in 18 clusters composed of 1,031 nodes (Fig. 6). Forty percent of these sequences were in cluster 1, which was composed exclusively of pyrrolysine-containing genes for directly utilizing TMA (*mttB*). Prior knowledge of this TMA-utilizing metabolism was limited to a study focused on available genomes within the *Bilophila* and a study of six draft methanogen genomes (24, 36). In comparison, MAGICdb contains 1,071 *mttB* genes assigned to *Bilophila* from multiple species and 61 methanogen genomes that span 3 genera, including one uncultivated genus UBA71 (Fig. 3A). Outside these lineages, we recovered 407 TMA-utilizing genomes (encode *mttB*) that were assigned to seven bacterial genera with additional representation from members of *Emergencia* and a novel genus in the Anaerovoracaceae (Fig. 6B).

We next sought to understand which of these lineages expressed genes for directly lowering TMA concentrations. Members of the *Biliophila*, *Emergencia*, and the methanogenic *Methanomassiliicoccus* were the most active in metatranscriptomes across the cohort. While a gene from a methanogenic *Methanomethylophilaceae* genome was the most highly transcribed (fourfold greater than the others) in a single sample, this gene was only found in 3% of samples. This is true in general for the methanogen TMA demethylating potential, while active in specific humans, it is sparsely distributed (Fig. 2E). The high level of activity of these methanogen and other members in certain humans, which would directly remove TMA from hepatic circulation, indicates how the personalized composition of the gut microbiota between individuals could be an underappreciated moderator of heart disease risk.

This study is the first inventory of genes that convert quaternary amines to non-TMA products (*mtxB*) in the human gut, cataloging 1,863 genes from 43 genera. More than half of the 17 non-pyrrolysine clusters contained a representative that was expressed *in vivo* or *in vitro*, while two of these clusters included six protein sequences that were previously experimentally verified to demethylate the quaternary amines studied here (Fig. 6). Of these non-TMA clusters, cluster 5 with 80% of the sequence diversity assigned to *Eubacterium* had the most representatives expressed in human cohorts (Fig. 5 and 6). Members of the genus *Bilophila* had the highest mean transcription across the cohort, and combined with the TMA-utilizing results, demonstrate an important, atherogenic-reducing role for this genus (Fig. 5). In conclusion MAGICdb recovered MA gene content previously unnoticed in prior microbiome publications, demonstrating the utility of this database to expedite the sampling of microbiome MA metabolism across wider ranges of humans and disease conditions.

### Gut microbiota markers predict cardiovascular disease in humans

While gut microbiota are commonly implicated in cardiovascular disease, the compilation of both proatherogenic and nonatherogenic genes that modulate gut TMA concentrations has not been systematically examined. Previous work sampled the proatherogenic genes that yielded TMA from choline (*cutC*) or carnitine (*cntA*/*yeaW*) in fecal metagenomes from 218 individuals with ACVD and 187 healthy controls. Using a database of 17 genes recovered in the study (34), this analysis failed to classify disease status in the cohort based on the relative abundance of these genes, with a cross-validation area under the curve (AUC) value of 0.63.

Here, we reanalyzed this metagenomic data set (34), but instead used the 3,031 unique genes in the MAGICdb for read recruitment (Fig. 3A). For context, MAGICdb has a 62- and 161-fold more sampling of *cutC* and *cntA*/*yeaW* gene richness, respectively, but also included the other MA genes not in the previous analysis (*grdI*, *mtxB*, *mttB*). Through read mapping MAGICdb uncovered 2,699 unique MA genes residing in these fecal metagenomes (Fig. S7). We showed that ACVD subjects had increased relative abundance of proatherogenic genes (*cutC, cntA, yeaW, grdI*). Additionally, while there was no significant difference of nonatherogenic mtxB relative abundance between AVCD and non-ACVD individuals, nonatherogenic *mttB* gene relative abundance was significantly depleted in AVCD subjects (Fig. S6).

**Fig 7:**
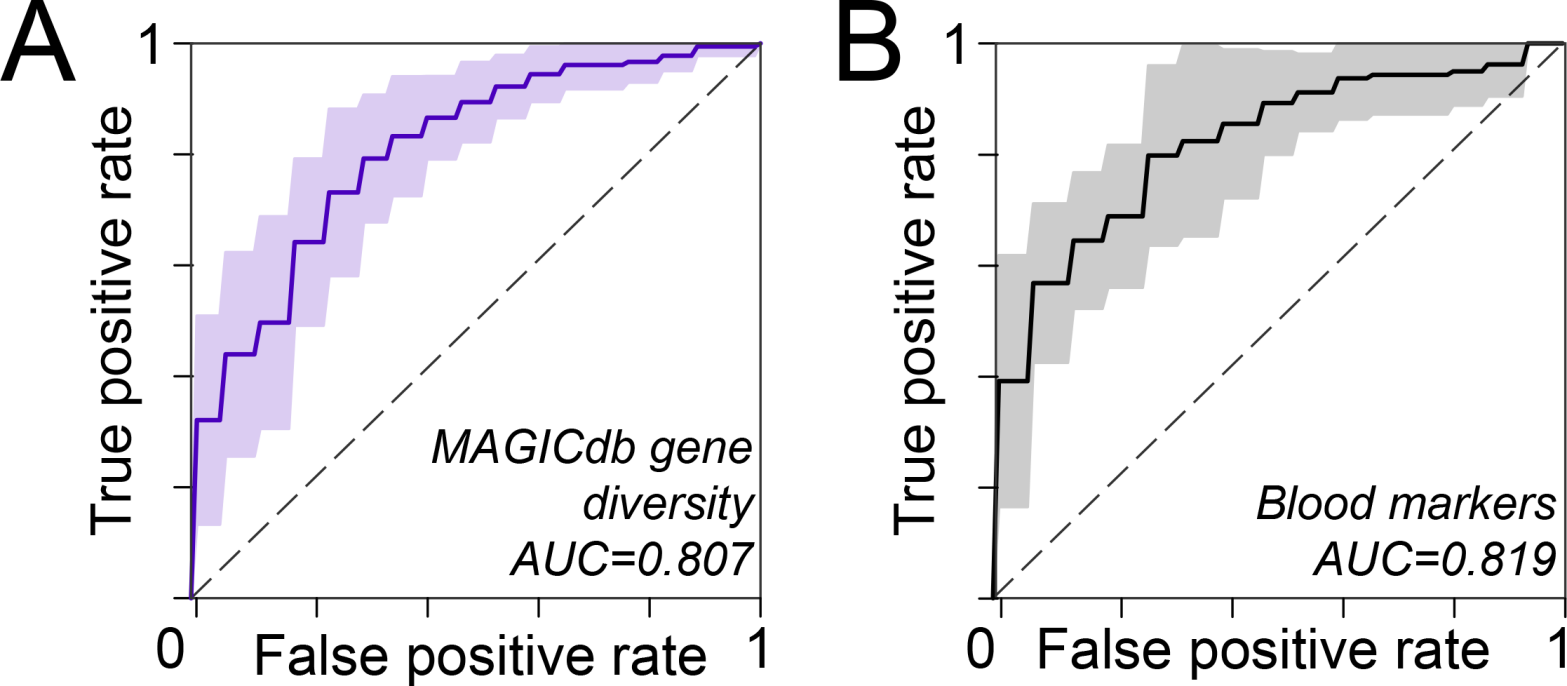
MAGICdb gene content predicted cardiovascular disease in humans. Logistic regression models were built using (**A**) richness and distribution of MAGICdb genes and (**B**) blood markers (LDL, HDL, and triglycerides) from a human cohort of 218 individuals with atherosclerotic cardiovascular disease and 187 healthy controls. Receiver operating curves from each model show the area under curve (AUC) for predictions of ACVD status in humans. These microbiome- and host-derived AUC were not significantly different (McNemar’s, *P*-value > 0.05).

To ascertain the enhanced prediction provided by the increased gene richness, the logistic regression model using only *cutC* and *cntA/yeaW* from MAGICdb had an AUC value of 0.67, with a slightly improved classification from the original model (Fig. S7). However, expanding our model to include the relative abundance for the full gene set (*cutC, cntA, yeaW, grdI, mtxB,* and *mttB*) and the diversity profile of these genes across the cohort (Fig. 7A) added to the predictability, resulting in AUC values of 0.75 and 0.81, respectively. These significantly increased values indicated the power of including both proatherogenic and nonatherogenic gene diversity when classifying ACVD health status. With an AUC of 0.81 these MA gene-based predictions did not differ significantly from predictions reliant on more traditional cumulative blood markers (HDL, LDL, triglycerides) in this same cohort (Fig. 7B). Collectively, our results establish the utility of metabolism-oriented microbiome databases to guide modern precision medicine strategies designed to correct defects in the gut microbiome.

### Conclusions

Future fecal metagenomic studies from larger cross-sectional cohorts of ACVD and healthy individuals are required to validate these MAGICdb-enabled disease predictions. However, these findings show the promise of this resource to streamline fecal microbiome analyses through (i) expertly curated annotations for these challenging gene sets and (ii) enhanced recovery of genes by read recruitment, a less computational and sequencing depth requiring process. In cohort studies there are tradeoffs between the number of individuals sampled and the sequencing depth of each sample. This commonly results in less sequencing depth, which constrains recovery and limits predictability of the gene data sets. This is especially relevant in cases such as MA metabolism where the functionality is encoded by rare, but active members of the gut community. We designed MAGICdb to alleviate this burden, providing an extensive curation of the proatherogenic and non-TMA-producing genes from cultivated and uncultivated lineages in the human gut microbiome. Ultimately, from a biomedical standpoint, MAGICdb provides an avenue for the direct comparison between microbial MA metabolism and disease outcomes in humans.

MAGICdb is the first comprehensive catalog of gut microbial MA metabolism, inventorying the thousands of genomes that encode the seven gene types responsible for mediating TMA gut concentrations. We leveraged this database with our own and other’s metagenome, metatranscriptome, and metaproteome data sets to show that these gut MA genes are active, coordinated, and predictive of cardiovascular disease. This open-access database and accompanying models can be applied to larger cohorts, opening the door for nontraditional microbiota tools for diagnosing, halting, and reversing cardiovascular disease. Additionally, this gene foundation can be exploited to discover the microbial MA contributions to other diseases, as this metabolism has been implicated in diabetes, as well as cerebral, hepatic, and vascular conditions (42, 43). Combined our case studies affirm MAGICdb as a valuable resource for the scientific community to further study the mechanistic details of cardiovascular disease and other diseases impacted by MA metabolism.

## MATERIALS AND METHODS

### MAGICdb construction and analysis

Combining the 1,436 MAGs recovered in this study with (i) 700 genomes from isolates in the Human Microbiome Project (HMP) (28) and (ii) 237,273 gut-derived MAGs from previously published studies (26, 27), we obtained 238,530 gut-associated genomes for analysis of MA metabolic potential. MAGs in group ii were compilation studies, where MAGs were accumulated across many publications representing many different lifestyles, disease types, and diets (26, 27). As outlined in Fig. S1, each gene type in Fig. 1 was assessed separately. First, using an experimentally validated amino acid sequence, each gene type was searched against the predicted amino acid sequences of the 238,530 gut-associated genomes using BLAST (44), retaining sequences with >60 bitscore. For CutC, CntA, YeaW, and GrdI, sequences were aligned with experimentally validated reference sequences using muscle, and phylogenetic trees were built using RAxML (45). Individual gene trees were visualized in iTOL (46), and the branch containing sequences of interest were selected. For the remaining sequences, active residues were confirmed as outlined for CutC, CntA, YeaW, and GrdI (11, 13, 14, 23). Of note are CntA and YeaW, which we report together as specificity cannot be inferred from sequence information alone (13, 14). The remaining sequences with active residues were then incorporated into MAGICdb, as well as their corresponding genomes into MAGICdb.

For MttB superfamily genes that did or did not contain pyrrolysine, a different approach was taken due to pyrrolysine interpreted as a stop codon during gene calling (20, 21). After recovery of putative MttB homologs using amino acid BLAST (44), obtained sequences were length filtered to 360 bp and aligned to known MttB superfamily members. Sequences longer than 360 did not contain pyrrolysine and aligned through the pyrrolysine residue were incorporated into the MAGICdb as non-pyrrolysine containing MtxB, as well as their corresponding genomes into the MAGICdb. These superfamily sequences could not be assigned a specific quaternary amine substrate, as such we denoted these as MtxB to indicate an unassigned substrate “X,” nomenclature consistent with the MttB superfamily (e.g., MtgB for glycine betaine [16], MtcB for carnitine [8]). The remaining truncated genes were then manually called in Geneious (47) from the original genome scaffolds using the amber read-through option to detect pyrrolysine. The resulting sequences that encoded for pyrrolysine were incorporated into the MAGIC gene database as pyrrolysinecontaining MttB, as well as their corresponding genomes into the MAGICdb.

MTTB superfamily genes in MAGICdb were used to construct a sequence similarity network via the EFI-EST web tool (48). Networks were generated with initial edge values of >80%, and sequences with 100% sequence similarity were collapsed into single nodes. The resulting representative node network was visualized with Cytoscape 3.8 (49) using the perfuse force-directed layout option (Fig. 6). Genomes in MAGICdb were analyzed with GTDB-Tk (50) for taxonomy, checkM (51) for quality, and DRAM (52) for genome annotation, with data reported in Data S1 and S2; Data S4 and S5 and Zenodo (see data availability statement).

### Sample procurement and cohort statistics

The current study considered samples collected from 125 individuals aged 21 years or older under the auspices of Dr. Alan George Smulian at either the University of Cincinnati College of Medicine or the University of Cincinnati Medical Center Holmes Hospital Outpatient Services. Each individual provided self-collected fecal, along with data on medical history (e.g., antibiotic usage, recent colonoscopy), weight, age, dietary habits, and smoking status (Data S1). Donor identities were stripped from the paired samples and their associated data, and each donor was assigned a unique identification number. Targeted metabolomic analyses of TMA were carried out on fecal samples from all 125 individuals, while a subset of 54 samples was selected based for fecal metagenomic sequencing along a fecal TMA gradient (Fig. S3). Based on surveys, subjects and their corresponding samples were removed from analyses due to antibiotic use in the last 6 months, lack of patient information, or a colonoscopy in the last 6 months, confining the cohort to 113 subjects. Five sets of donated samples were removed from analyses due to donor antibiotic use and seven were removed for lack of donor de-identified data. Written, informed consent was obtained from all study participants, and subject treatment and experiments with donated samples were approved by Institutional Review Boards of the University of Cincinnati and the Ohio State University.

### Metagenomic sequencing, assembly, and binning for this cohort and MA reactors

Fifty-four fecal samples out of 113 were chosen across a TMA gradient for metagenomic sequencing, with at least five samples chosen from each quartile (Fig. 2B). Total nucleic acids were extracted from five microcosm samples and 54 human fecal samples using the PowerSoil DNA Isolation kit (MoBio), eluted in 100 µL, and stored at −20°C until sequencing. DNA was submitted for sequencing at the Genomics Shared Resource facility at The Ohio State University. Libraries were prepared with the Nextera XT Library System in accordance with the manufacturer’s instructions. Genomic DNA was sheared by sonication, and fragments were end-repaired. Sequencing adapters were ligated, and library fragments were amplified with five cycles of PCR before solid-phase reversible immobilization size selection, library quantification, and validation. Libraries were sequenced on the Illumina HiSeq 2500 platform and paired-end reads of 113 cycles were collected. All raw reads from microcosms and fecal samples were trimmed from both the 5´ and 3´ ends with Sickle (https://github.com/najoshi/sickle), and then each sample was assembled individually with IDBA-UD (53) using default parameters. Metagenome statistics including amount of sequencing are noted in Data S1.

All microcosm and fecal metagenomes (Data S1) were binned using metabat2 (54) with default parameters. Bins were then assessed for quality using checkM (51). Metagenomic reads from the binned samples were then mapped to bins >50% completion and <10% contamination (medium- or high-quality bins [55] at 99% identity using bbmap [56]). For deeply sequenced metagenomes (*n* = 15) reads that did not map to the pool of medium- or high-quality bins were then reassembled using IDBA-UD (53), completing iterative assemblies for each of the 15 samples, until no new bins could be recovered. The resulting 2,447 bins were then dereplicated into 1,436 bins using dRep (57).

### Fecal metabolite analyses from the cohort study

Fecal samples were self-collected by volunteers and brought to the collection center where they were stored at −80°C. Samples were then shipped to the lab for analysis on dry ice where they were again stored. Samples arrived frozen in less than 24 h and were immediately stored at −80°C until ready for NMR analysis.

Fecal samples were removed from the freezer and transferred to a biosafety cabinet on dry ice. A total of 0.2 to 0.5 g (wet weight) of frozen chips of each sample were weighed and transferred to a 5 mL centrifuge tube. To extract metabolites from the fecal samples, 1 mL 0.75 M potassium phosphate buffer (PBS buffer) in 50% D2O, pH 7.2, was added to each tube, resulting in either 3× (vol/wt) dilution (for fecal samples with more than 0.3 g in wet weight) or 5× (vol/wt) dilution (for fecal samples with less than 0.3 g in wet weight) of the original samples. The slurries were then vortexed for a total of 3 min to extract metabolites. Vortexing was paused several times in order to cool the sample on ice to avoid overheating. The vortexed samples were then centrifuged at 1000 × *g* for 10 min at 4°C. The supernatant was transferred to a 1.5-mL microcentrifuge tube and were centrifuged again twice at 4°C (16,100 × *g*, 10  min) to remove remaining debris. Total 200 µL of final supernatant were mixed with 100 µM DSS and transferred to a 3 mm × 178 mm NMR tube for NMR analysis.

1D ^1^H and 2D ^1^H-^13^C HSQC NMR spectra were conducted at 298 K on a Bruker Avance III HD 800 MHz (Billerica, MA) at Ohio State campus chemical instrument center (CCIC) NMR facility. Proton NMR, about 4 min for one data set, was acquired using 1.28 s acquisition time, 2 s relaxation delay, and 64 number of scans. The water suppression was achieved using excitation sculpting with gradients. 2D ^1^H-^13^C HSQC was acquired with a standard Bruker pulse sequence using phase-sensitive echo/antiecho-TPPI gradient selection. The experiment parameters include ~4 ms acquisition time in ^13^C dimension, ~80 ms acquisition time in ^1^H dimension, 1 s relaxation delay, 16 number of scans, ^13^C GARP decoupling during acquisition, and data matrix of 2048 × 128. The experimental time is roughly 38 min for one data set. Standards with 100 µM of target metabolites (>98% purify) were analyzed under the same conditions. When appropriate, sample aliquots were spiked with a known concentration of TMA to confirm peak assignment.

All NMR data were processed with Bruker Topspin 3.6.1 (Billerica, MA). The data were typically zero-filled one time in both ^1^H and ^13^C dimension prior to the application of window functions, followed by Fourier transformation, phasing, and baseline correction. Chemical shifts were internally referenced to DSS at 0.00 ppm. The concentration of a TMA was estimated employing standards of known concentration and comparing the integral of peaks to DSS.

### Cohort analyses

We leveraged our cohort metagenomes to understand the distribution of MA genes with variable depths of sequencing and in relation to fecal TMA concentrations. First, we mined our fecal metagenome assemblies for MA genes, finding 153 MA genes that were dereplicated into 135 genes using cd-hit (58). We grouped subjects into quartiles (Q1–Q4, 25% of the data points in each) and then related the paired gene content as shown in Fig. 2B. To understand the recovery of new genes with additional subjects and sequencing, we performed a species accumulation analysis where genes recovered from each metagenome were iteratively dereplicated with the addition of each subject using cd-hit (58), as shown in Fig. 2C. To obtain gene abundance, we mapped metagenomic reads rarified to 8 Gbp of sequencing to the dereplicated gene set (*n* = 135) using bowtie2 (59). Reads were counted and summarized using coverM (https://github.com/wwood/CoverM) into trimmed mean (-m trimmed_mean) and including genes with a minimum covered fraction of 75% (--min-covered-fraction 0.75). To relate gene abundance to fecal TMA concentration, we used linear regression-based modeling to predict TMA concentrations from MA gene-relative abundance in our cohort using sparse partial least squares [sPLS (60, 61)] as implemented in the R package mixOmics (62), with data shown in Fig. S3D. To further understand how gene recovery was impacted by sequencing depth, we used our most deeply sequenced metagenomes (>35 Gbp) to recruit reads to the cohort gene database (*n* = 135) using all reads for a particular metagenome and reads rarified to 4 Gbp from the same metagenome, with gene abundance and gene count reported in Fig. S3C. Briefly, we mapped all metagenomic reads or reads rarified to 4 Gbp [similar to previous depths used in other microbiome studies (29, 34) of (*n* = 135) using bowtie2 (59)]. Reads were counted and summarized using coverM (https://github.com/wwood/CoverM) into trimmed mean (-m trimmed_mean) and including genes with a minimum covered fraction of 75% (--min-covered-fraction 0.75).

Beyond the gene level, within our cohort, we aimed to understand the distribution of MA genomes in the context of the microbial community. Abundance data reported was based on the 1,436 unique bins. Briefly, reads from metagenomes with greater than 8 Gbp in depth were rarified to 8 Gbp from all 52 metagenomes and mapped to 1,436 unique bins using bowtie2 (59) with 95% identity and counted using coverM (https://github.com/wwood/CoverM) within trimmed mean mode (-m trimmed_mean) and including genomes with a minimum covered fraction of 75% (--min-covered-fraction 0.75). Trimmed mean values were then transformed into relative abundance. To determine rank of each genome, relative abundance of each genome was averaged across the cohort and then ordered from maximum to minimum, and ranks were assigned 1–1,436, as shown in Fig. 2D and E. Note, only 52 metagenomes were used in this analysis, as two were dropped due to sequencing <8 Gbp.

### MA reactor construction and operation

The microcosm experiment consisted of six treatments all set up with fecal material from subject 74: (i) no substrate and fecal material, (ii) glycine betaine and fecal material, (iii) carnitine and fecal material, (iv) butyrobetaine and fecal material, and (v) choline and fecal material. Each treatment was done in triplicate and consisted of 10% (wet weight/volume) anoxic, fecal slurry in sterile basal bicarbonate-buffered medium dispensed in Balch tubes sealed with butyl rubber stoppers and aluminum crimps under an atmosphere of N_2_/CO_2_ (80:20 [vol/vol]), with a final volume of 10 mL. Before mixing with fecal slurry, the medium (per liter) included 0.25 g ammonium chloride, 0.60 g sodium phosphate, 0.10 g potassium chloride, 2.5 g sodium bicarbonate, 10  mL dl-vitamin mixture, and 10  mL dl-mineral mixture and was brought to a pH of 7.0 using 1  mM NaOH (63). Tubes were incubated at 37°C. Samples for metagenomics and metaproteomics were taken at the final (T_F_) timepoint, while metabolite samples were taken at the indicated times during the course of the 25 d incubation (Fig. 4A). Anoxic fecal reactors were primed with 40 µM of each substrate from time of inoculation to day 3, then they were dosed with 1 mM of each substrate three times at day 3, day 10, and day 17. Accounting for removal of 1 mL samplings, a total of 27 µmol of each substrate was added. Samples were taken for subsequent analysis at T_1_ (10 days), T2 (17 days), and T_F_ (25 days). For timepoints T_1_ and T2, samples were taken prior to substrate addition. Subject 74 fecal material, used for reactor inoculum, TMA concentrations are given in Data S1.

### MA reactor metabolomic data acquisition and analysis

Samples from microcosm experiments were filtered (0.2 µm) at time of collection and sent to the Pacific Northwest National Laboratory for metabolite analysis by NMR. Samples were diluted by 10% (vol/vol) with 5 mM 2,2-dimethyl-2-silapentane-5-sulfonate-d_6_ as an internal standard. All NMR spectra were collected using a Varian Direct Drive 600-MHz NMR spectrometer equipped with a 5 mm triple resonance salt-tolerant cold probe. The 1D ^1^H NMR spectra of all samples were processed, assigned, and analyzed using Chenomx NMR Suite 8.3 with quantification based on spectral intensities relative to the internal standard. Candidate metabolites present in each of the complex mixtures were determined by matching the chemical shift, J-coupling, and intensity information of experimental NMR signals against the NMR signals of standard metabolites in the Chenomx library. The 1D ^1^H spectra were collected following Chenomx data collection guidelines (64), using a 1D NOESY presaturation (TNNOESY) experiment with at least 512 scans at 298K using a 100 ms mixing time, with 12 ppm spectral width, a 4-s acquisition time followed by a relaxation delay of 1.5 s during which a presaturation of the water signal applied. Post-acquisition processing included time domain-free induction decays (57,472 total points) zero-filling to 132 k points and multiplication by a decaying exponential function (line broadening of 0.5 Hz) prior to Fourier transform. Chemical shifts were referenced to the ^1^H methyl signal in DSS-d_6_ at 0 ppm. Additionally, 2D spectra (including ^1^H–^13^C heteronuclear single-quantum correlation spectroscopy, ^1^H^–1^H total correlation spectroscopy) were acquired on a subset of the fluid samples. Biological triplicates had similar metabolite pools, with all data reported (Data S3).

### MA reactor metaproteomic extraction, spectral analysis, and data acquisition

Liquid culture (1.2 mL) from each microcosm sample was collected anaerobically, centrifuged for 15 min at 10,000 × *g*, separated from the supernatant, and stored at −80°C until shipment to Pacific Northwest National Laboratory. Proteins in the pellet were precipitated and washed twice with acetone. Then the pellet was lightly dried under nitrogen.

Each precipitated protein pellet was diluted in 200 µL of 8 M urea in 100 mM ammonium bicarbonate, pH 8 (ABC). A bicinchoninic acid (BCA) assay (Thermo Scientific, Waltham, MA, USA) was performed to determine protein concentration. Following the assay, 10 mM dithiothreitol (DTT) was added to the samples and incubated at 60°C for 30 mins with constant shaking at 800 rpm. Samples were then diluted eightfold for preparation for digestion with 100 mM ABC, 1 mM CaCl2, and sequencing-grade modified porcine trypsin (Promega, Madison, WI, USA) was added to all protein samples at a 1:50 (wt/wt) trypsin-to-protein ratio for 3 h at 37°C. Digested samples were desalted using a four-probe positive pressure Gilson GX-274 ASPEC system (Gilson Inc., Middleton, WI, USA) with Discovery C18 50 mg/1 mL solid-phase extraction tubes (Supelco, St. Louis, MO, USA), using the following protocol: 3 mL of methanol was added for conditioning followed by 2 mL of 0.1% TFA in H2O. The samples were then loaded onto each column followed by 4 mL of 95:5: H2O:ACN, 0.1% TFA. Samples were eluted with 1 mL 80:20 ACN:H2O, 0.1% TFA. The samples were concentrated down to ~100 µL using a Speed Vac and a final BCA was performed to determine the peptide concentration and samples were diluted to 0.1 µg/µL with nanopure water for MS analysis.

All mass spectrometric data were acquired using a Q-Exactive Plus (Thermo Scientific) connected to a nanoACQUITY UPLC M-Class liquid chromatography system (Waters) via in-house 70 cm column packed using Phenomenex Jupiter 3 µm C18 particles and in-house-built electrospray apparatus. MS/MS spectra were compared with the predicted protein collections using the search tool MSGF+ (65). Contaminant proteins typically observed in proteomics experiments were also included in the protein collections searched. The searches were performed using ±20 ppm parent mass tolerance, parent signal isotope correction, partially tryptic enzymatic cleavage rules, and variable oxidation of methionine. In addition, a decoy sequence approach (66) was employed to assess false-discovery rates. Data were collated using an in-house program, imported into a SQL server database, filtered to ∼1% false-discovery rate (peptide to spectrum level), and combined at the protein level to provide unique peptide count (per protein) and observation count (i.e., spectral count) data. Spectral count data for each identified protein was normalized using normalized spectral abundance frequency (NSAF) calculations (67, 68), accounting for protein length and proteins per sample (Data S3). Note that metaproteomics were not done on raw fecal samples. Metaproteomes were mapped to dereplicated MAGICdb-predicted amino acid sequences, as well as predicted amino acid sequences of unique MAGs recovered from enrichments. Of note, the recently discovered *bbu* gene cluster (69, 70) that converts butyrobetaine to TMA under anaerobic conditions and the organism in which it was characterized (*Emergencia timonensis*) was below detection in our reactor metaproteomes, further confirming the nonatherogenic response detected in carnitine and butryobetaine reactors.

### Mapping of published data to MAGICdb

All reads were downloaded from EBI from Abu-Ali et al.’s (40) study of metatranscriptomes from adult men. Adapters were stripped using bbduk.sh with the parameters ktrim = r, k = 23, mink = 11, hdist = 1. Reads were trimmed using sickle with default parameters. Reads were mapped to MAGICdb genes using bbmap.sh [bbtools suite (56) using perfectmode = t and ambiguous = random]. Counts were extracted from the bbmap covstats output and compiled into a table. The counts were then transformed to geTMMs (71).

All proteome .mgf files were downloaded from Lloyd-Price et al. (41). Files were then searched against the MAGICdb using MSGF+ (65) using the parameters inst 3, tda 1, ti 1,3, ntt 1, and maxLength 50. After the search files were converted to TSVs using the parameter showDecoy 1. To determine hits, first all hits with a pep q-value >0.01 were removed. Then for each sample we identified proteins with more than one peptide hit. This list of proteins per sample were the ones considered present.

### CVD prediction from human gut metagenomic data

All reads were downloaded from EBI from Jie et al.’s (34) study of metagenomes from 218 individuals with ACVD and 187 healthy controls (15). Adapters were stripped using bbduk.sh with the parameters ktrim = r, k = 23, mink = 11, hdist = 1. Reads were trimmed using sickle with default parameters. Reads were mapped to unique MA genes in MAGICdb genes using bbmap.sh (bbtools suite [56] using perfectmode = t and ambiguous = random). Counts were extracted from the bbmap covstats output and compiled into a table. The counts were then normalized to geTMM, a gene length-corrected (ge) trend means of m-values (TMM), which is a method for assessing intrasample variation for read map data (71). For each individual we obtained the relative abundance profile of the MA genes (*cutC, cntA, yeaW, grdI, mtxB,* and *mttB*).

The relative abundance MA gene profiles were then used in a logistic regression model using scikit-learn (72) to predict ACVD status (0 = No ACVD, 1 = ACVD) as designated in Jie et al. (34). Models were evaluated using stratified 10-fold cross-validation with mean false-positive and true-positive rates reported and used to calculate the area under the receiver operator characteristic curve (AUC-ROC) (73). Feature coefficients for logistic regression model for the best-performing model (Shannon’s diversity of each type of gene in MAGICdb) were reported. Genders included in the models were noted and dichotomized so that male equals one and female equals 0. An AUC value >0.7 was used to indicate a relatively good ability for the model to classify individual disease status (74). To test for difference in model performance McNemar’s test was used (75).

Models were trained on the following to predict ACVD status:

- Shannon’s diversity of MAGICdb genes by gene type +gender (Fig. 7A). Shannon diversity score was determined for each gene type using the scikit-bio (http://scikit-bio.org/) and calculated using the geTMMs. Each individual had a Shannon’s diversity profile that included 6 MA gene diversity scores per individual.
- Blood markers (triglyceride mmol/L, LDL mmol/L, and HDL mmol/L) +gender (Fig. 7B)]
- Abundance of *cutC*, *cntA/yeaW* summed per gene type +gender [genes used and model analysis similar to reported in Jie et al. (34) (Fig. S7A)]
- Abundance of all genes from MAGICdb +gender (Fig. S7B), each gene abundance in the unique MAGICdb gene database is included.
- Abundance summed per gene type +gender (Fig. S7C)
- Abundance of all genes summed per atherogenic status (proatherogenic and nonatherogenic) +gender (Fig. S7D).
- Shannon’s diversity of MAGICdb genes by gene type (no gender) (Fig. S7E). Similar to above in number one but did not include gender.

## Data Availability

All MAGICdb files are available on Zenodo at doi Zenodo https://doi.org/10.5281/zenodo.7409848. Processed MAGs and raw NMR data for the 54 person cohort and methylated amine enrichments from this study are available in Zenodo at https://doi.org/10.5281/zenodo.7409994, with corresponding metagenomic reads deposited at NCBI under BioProject PRJNA725020. Metaproteomic data from laboratory reactors are deposited in the PRIDE database at ftp://massive.ucsd.edu/MSV000087004/.

## SUPPLEMENTAL MATERIAL

Data S1 mbio.01511-23-s0001.xlsx Cohort statistics, metabolite concentrations, and microbial community data statistics.

Data S2 mbio.01511-23-s0002.xlsx MAGICdb entries, including genome, taxonomy, and gene type.

Data S3 mbio.01511-23-s0003.xlsx Microcosm multi-omic data.

Data S4 mbio.01511-23-s0004.txt MAGICdb entries, paired to Data S2.

Data S5 mbio.01511-23-s0005.txt DRAM summary annotation file for genomes in MAGICdb.

Supplemental Text mbio.01511-23-s0006.docx Supplemental figures and legends.

ASM does not own the copyrights to Supplemental Material that may be linked to, or accessed through, an article. The authors have granted ASM a non-exclusive, world-wide license to publish the Supplemental Material files. Please contact the corresponding author directly for reuse.
