# Supplementary material for: Targeted curation of the gut microbial gene content modulating human cardiovascular disease
Source: mBio. 2023 Sep 11;14(5):e01511-23. doi: 10.1128/mbio.01511-23 (PMC10653893; doi:10.1128/mbio.01511-23)
Supplement: Supplemental Text — Supplemental figures and legends. [file mbio.01511-23-s0006.docx]

**
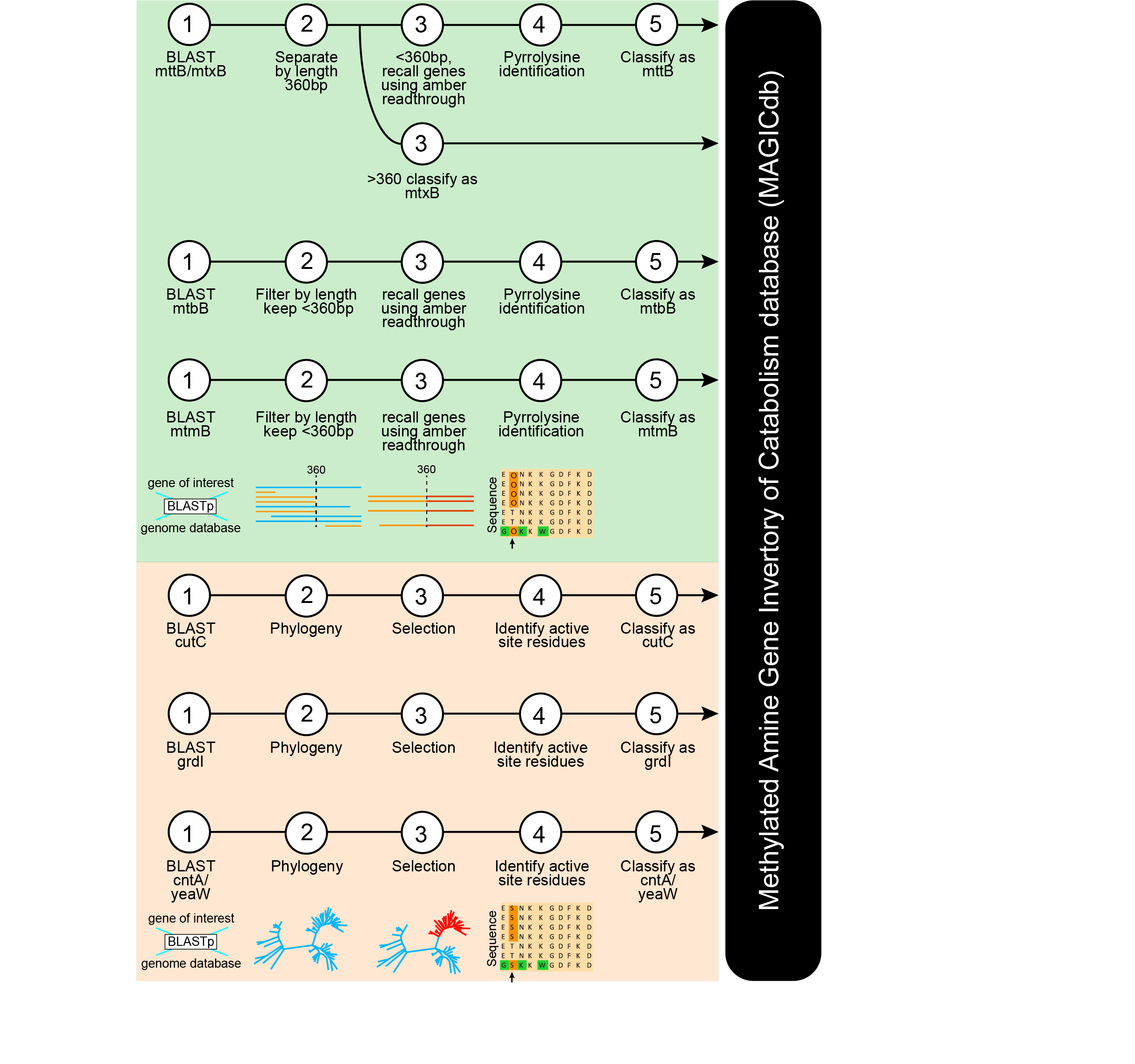
**

**Figure S1. Computational workflow for MAGICdb.** Our pipeline uses both homology and nonhomology based approaches to refine the annotation of MA gene content, with methods for annotation of gene function for methyltransferase reactions (top, green) and TMA producing reactions (bottom, orange) denoted. Details can also be found in methods section.


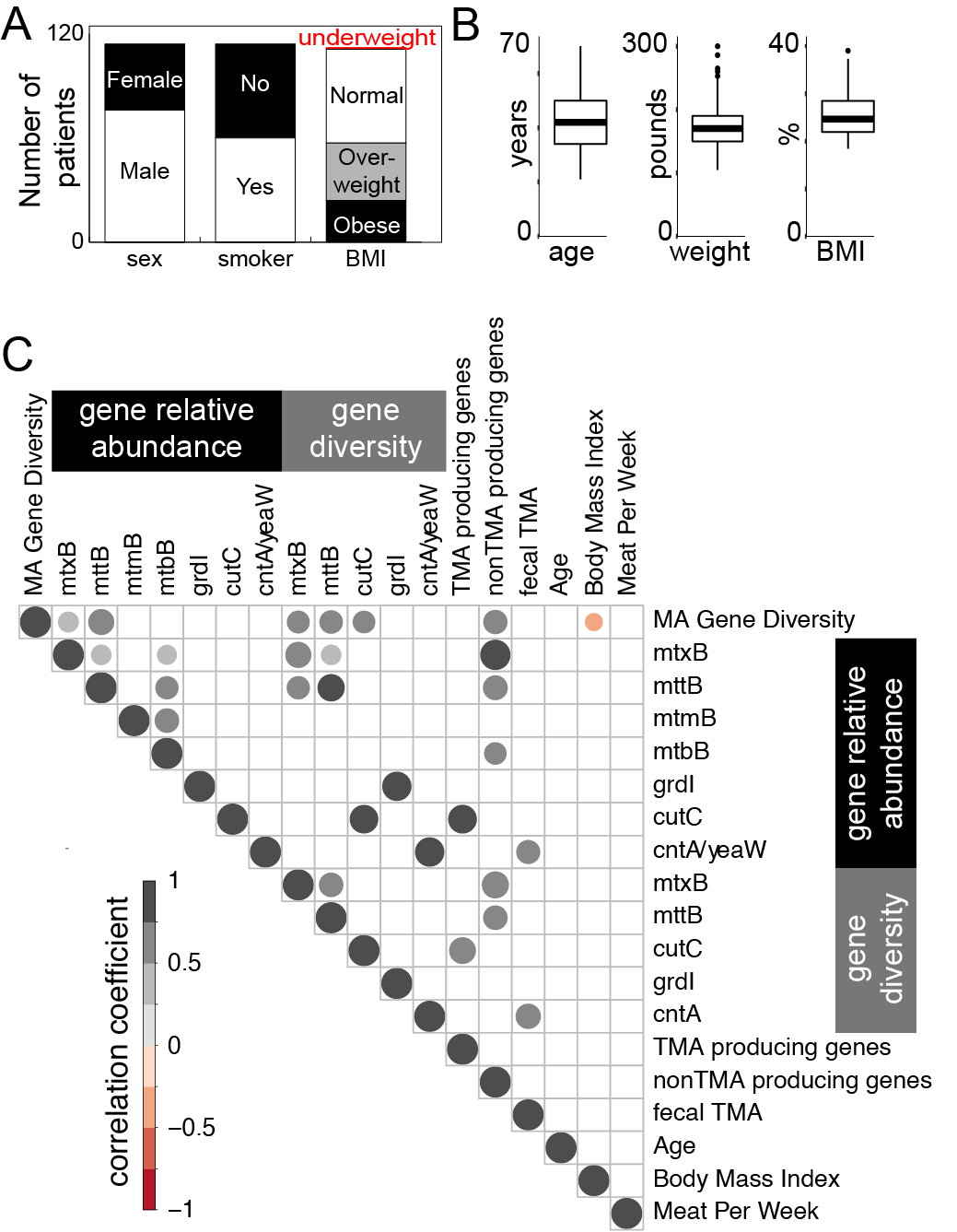


**Figure S2. Human cohort statistics.** **A** Cohort statistics including sex, smoking status, and BMI category of 113 human subjects. **B** The median values of age, weight, and BMI statistics across the cohort (n=113). Points above or below boxplots signify outliers, or values outside one standard deviation of the median. **C** Dot plot shows the all-to-all correlations of MA gene diversity, MA gene abundance, and host lifestyle factors, with the significant (p-value<0.01) correlations shown by dots colored and sized by correlation coefficients.


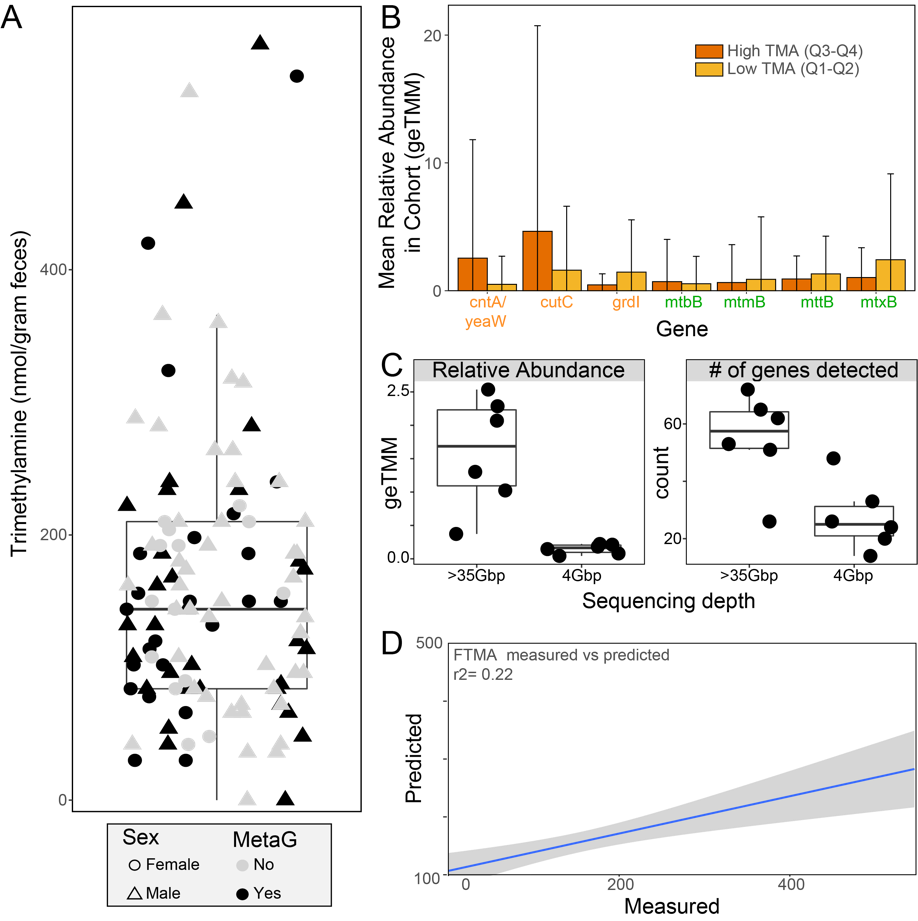


**Figure S3. Human cohort study reveals relationship between fecal trimethylamine concentrations and MAGICdb gene abundances. A** Fecal TMA concentrations, with color denoting the samples sequenced with metagenomics (n=54, black) and those not chosen (gray). Shape indicates the sex of the subject from which the fecal sample was derived. **B** Rarified metagenomes (8Gbp) were mapped to a database of 135 dereplicated MA genes to provide the mean relative abundance of each gene type across the cohort. The mean and standard deviation of the relative abundance of MA genes recovered from high (>145 nmol TMA/gram feces) and low (0-144 nmol TMA/gram feces) fecal TMA samples. **C** Comparison of the relative abundance (right) and count of MA genes recovered when >35Gbp and 4Gbp of reads were evaluated. **D** Prediction of fecal TMA from MA gene content using sparse Partial Least Squares (sPLS) regression revealed a significant relationship between the MA gene content predicted and measured fecal TMA concentrations, explaining 22% of the variability in TMA concentrations across the cohort.


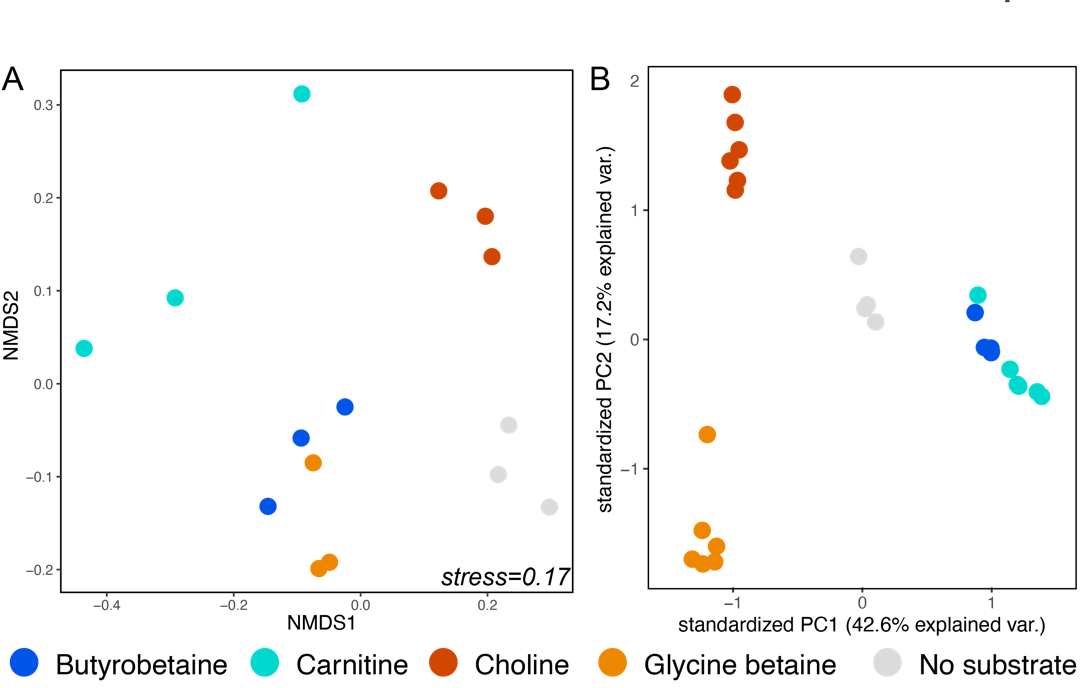


**Figure S4.** **Quaternary amine fed fecal reactors show distinct activity and chemical profiles relative to no substrate addition reactors (control).** Ordinations show community wide gene expression (**A**, non-metric multidimensional scaling of final timepoints only) and metabolome (**B**, principal component analysis of T2 and TF timepoints) profiles for each reactor, with sample points colored by quaternary amine addition. Reactor microbial communities are statistically different by treatment and timepoint (mrpp, p<0.001); while 42% and 17.2% of variance in the metabolome is explained by the first and second components.

**
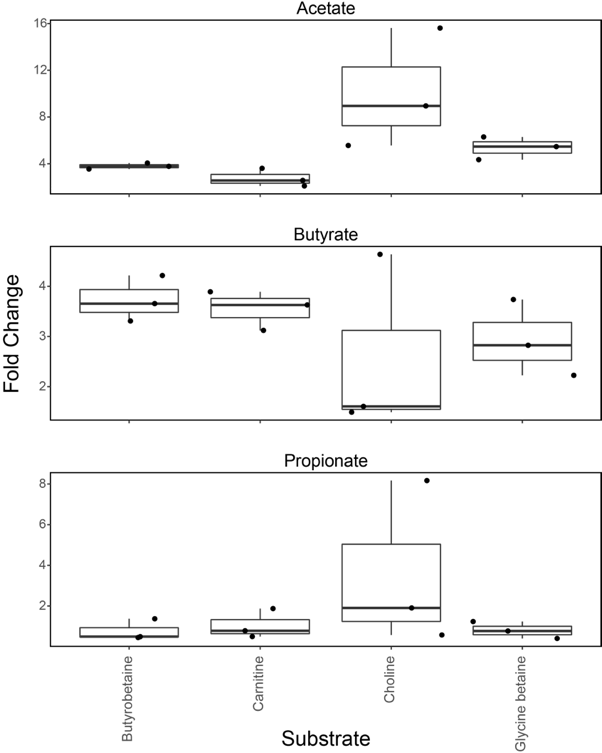
**

**Figure S5.** **Short chain fatty acid profiles of reactors reveal significant differences relative to no substrate controls at final time point.** Fold change in SCFA concentrations detected in final timepoint of MA gut reactors relative to no substrate controls, with boxplots representing the triplicate reactor concentration for each reactor substrate. SCFA production gene expression is given in **Data S3**.

**
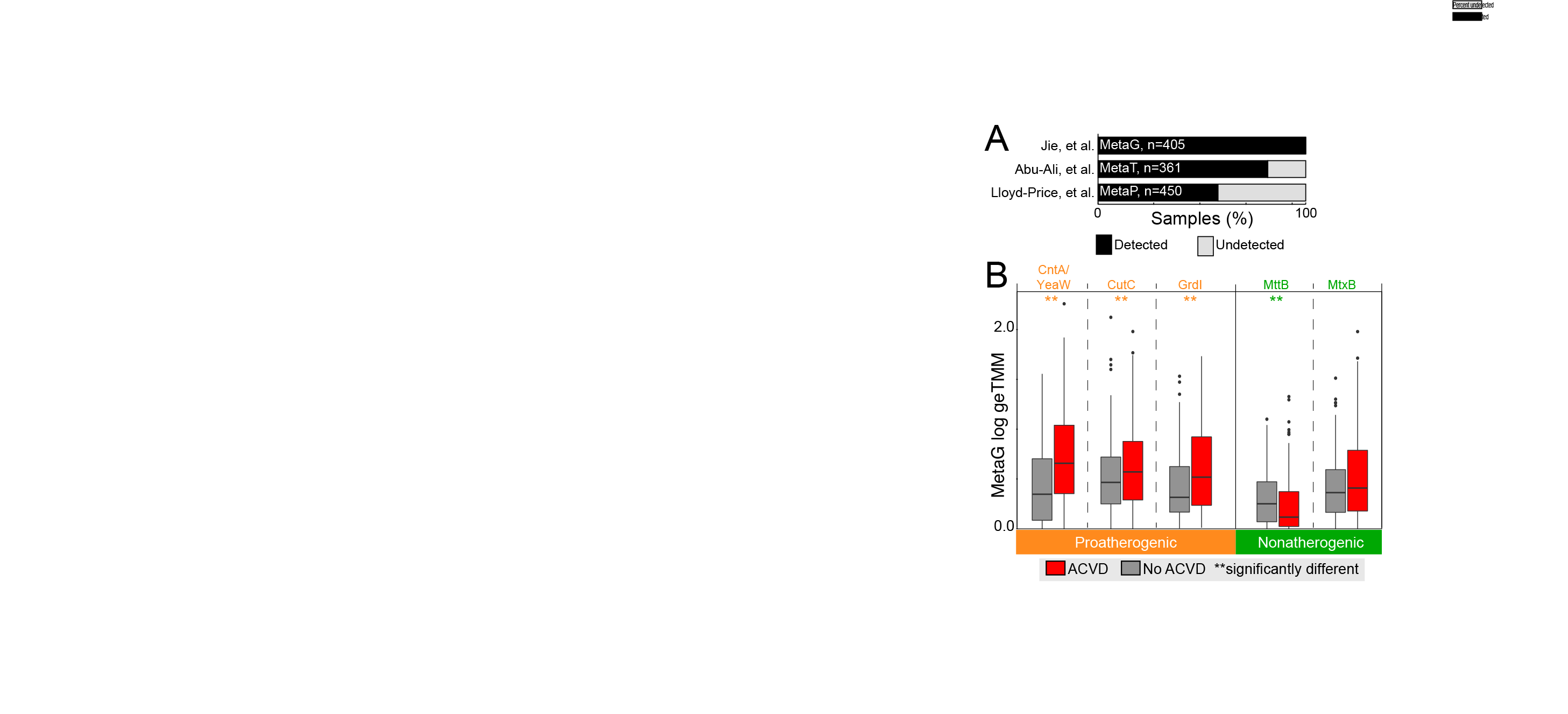
**

**Figure S6. Mapping of publicly available human expression data to MAGICdb uncovered previously enigmatic methylated amine functions in the gut microbiome. A** Bar chart denotes the percentage of samples per study that members of MAGICdb are present or active. Studies include metagenomic data from a cohort of 218 individuals with atherosclerotic cardiovascular disease and 187 healthy controls, metatranscriptomic data from 361 adult men, and metaproteomic data from longitudinal sampling of 132 patients with irritable bowel syndrome. **B** Boxplots display the relative abundance of MAGICdb genes in fecal metagenomes from ACVD patients (red) or non-ACVD control subjects (grey), with significant differences by ACVD status denoted by double asterisk.


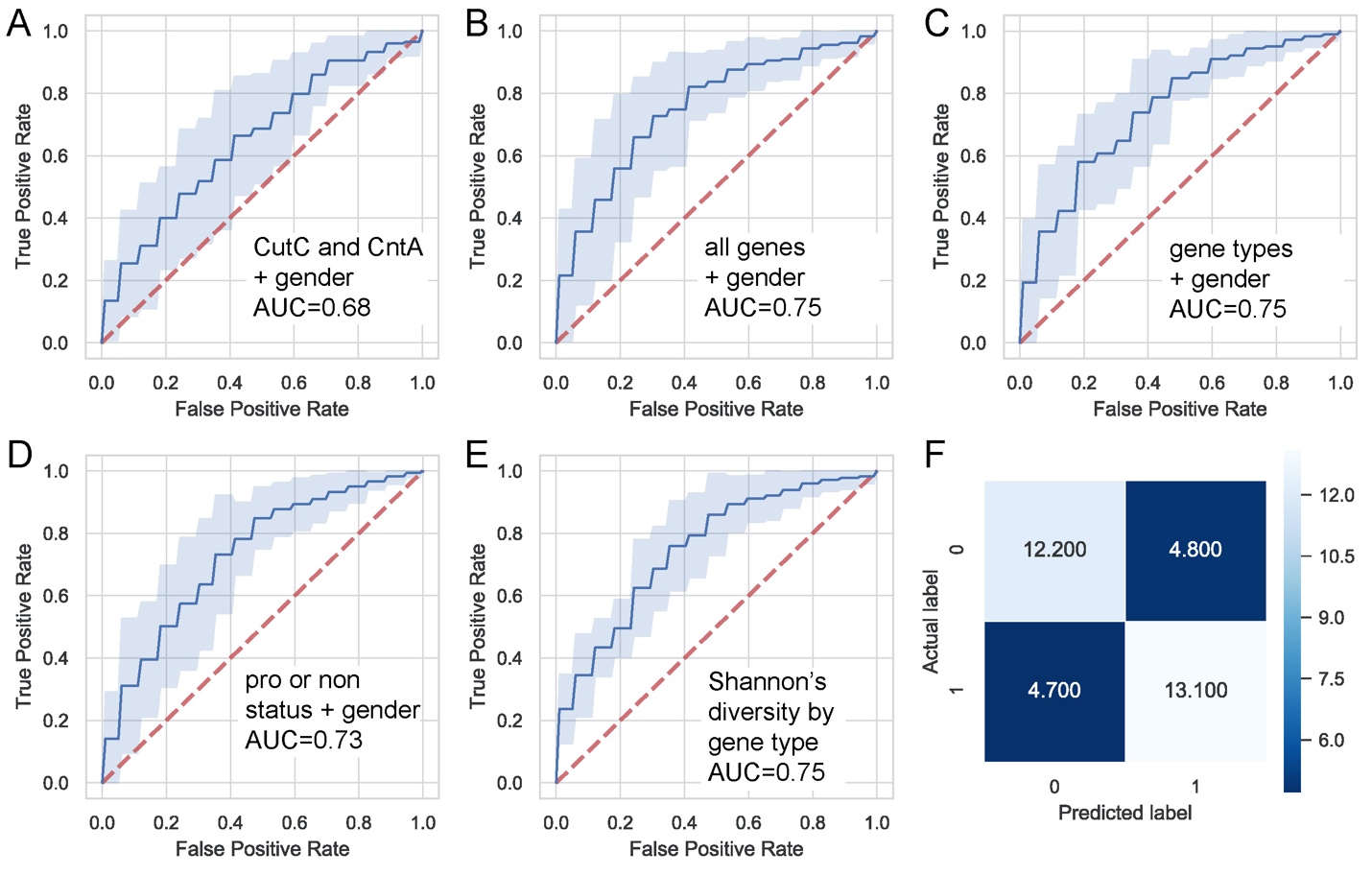


**Figure S7. Receiver operating curves (ROC) highlight the ability of MAGICdb content to enable prediction of ACVD in a human cohort from microbial gene content.** Logistic regression models were built using **A** abundance of *cutC*, *cntA/yeaW* summed per gene type + gender (genes used and model analysis similar to reported in ^34^, **B** abundance of all genes from MAGICdb + gender, each gene abundance in the unique MAGICdb gene database is included, **C** Abundance summed per gene type + gender, **D** Abundance of all genes summed per atherogenic status (proatherogenic and nonatherogenic) + gender, and **E** Shannon’s diversity of MAGICdb genes (no gender). **F** Confusion matrix for logistic regression model built based on the Shannon’s diversity of each type of gene in MAGICdb. Values are averaged over results from 10-fold cross validation.

**Supplementary File Legends**

**Data S1 (separate file).** Excel file containing cohort statistics, metabolite concentrations, and microbial community Data Statistics.

Tab 1) List of genes inventoried in MAGICdb including abbreviations, gene names, reactions, and citations.

Tab 2) Metagenome sequencing information including size (Gbp) reads, TMA concentrations, and number of MA genes recovered.

Tab 3) Cohort statistics including sex, age, weight, BMI, smoking status, meat consumption, and TMA concentrations.

Tab 4) Mapping for figure S3C mapping

Tab 5) MAG quality and taxonomy information recovered from human gut metagenome samples in this study.

Tab 6) Figure 2DE MA genes presence across cohort.

Tab 7) Genome relative abundance within the cohort, rarified to 8Gbp of sequencing depth.

**Data S2 (separate file)**. Excel file of MAGICdb entries, including genome, taxonomy, and gene type.

**Data S3 (separate file).** Microcosm multi-omic data.

Tab 1) Metaproteomic data.

Tab 2) Metabolite data obtained by NMR.

Tab 3) Gene corresponding to energy generation strategies and SCFA production with proteome expression across samples given.

**Data S4 (separate file)**. Fasta file of MAGICdb entries, paired to **Data S2**.

**Data S5 (separate file).** DRAM summary annotation file for genomes in MAGICdb.
